# Bay41-4109-induced aberrant polymers of hepatitis b capsid proteins are removed via STUB1-promoted p62-mediated macroautophagy

**DOI:** 10.1371/journal.ppat.1010204

**Published:** 2022-01-14

**Authors:** Jiacheng Lin, Limin Yin, Xia-Zhen Xu, He-Chen Sun, Zhi-Hua Huang, Xue-Yun Ni, Yan Chen, Xu Lin

**Affiliations:** 1 Key Laboratory of Gastrointestinal Cancer (Fujian Medical University), Ministry of Education, Fuzhou, China; 2 Fujian Key Laboratory of Tumor Microbiology, Department of Medical Microbiology, Fujian Medical University, Fuzhou, China; Albany Medical College, UNITED STATES

## Abstract

The hepatitis B virus (HBV) core protein (HBc) functions in multiple steps of the viral life cycle. Heteroaryldihydropyrimidine compounds (HAPs) such as Bay41-4109 are capsid protein allosteric modulators that accelerate HBc degradation and inhibit the virion secretion of HBV, specifically by misleading HBc assembly into aberrant non-capsid polymers. However, the subsequent cellular fates of these HAP-induced aberrant non-capsid polymers are not well understood. Here, we discovered that that the chaperone-binding E3 ubiquitin ligase protein STUB1 is required for the removal of Bay41-4109-induced aberrant non-capsid polymers from HepAD38 cells. Specifically, STUB1 recruits BAG3 to transport Bay41-4109-induced aberrant non-capsid polymers to the perinuclear region of cells, thereby initiating p62-mediated macroautophagy and lysosomal degradation. We also demonstrate that elevating the STUB1 level enhances the inhibitory effect of Bay41-4109 on the production of HBeAg and HBV virions in HepAD38 cells, in HBV-infected HepG2-NTCP cells, and in HBV transgenic mice. STUB1 overexpression also facilitates the inhibition of Bay41-4109 on the cccDNA formation in *de novo* infection of HBV. Understanding these molecular details paves the way for applying HAPs as a potentially curative regimen (or a component of a combination treatment) for eradicating HBV from hepatocytes of chronic infection patients.

## Author summary

Hepatitis B virus (HBV) infects more than 250 million people worldwide chronically. It is a major pathogen causing liver cirrhosis and hepatocellular carcinoma now. The HBV capsid protein (HBc) plays multiple roles in the viral life cycle, and many antivirals targeting HBc such as Heteroaryldihydropyrimidine compounds (HAPs) are under clinical trial recently. This study aimed to investigate how a HAP compound Bay41-4109 induces the degradation of HBc protein. Bay41-4109 induces aberrant non-capsid polymers, which form in complex with the chaperone-binding E3 ubiquitin ligase protein STUB1 and co-chaperone BAG3 and are transported to the perinuclear compartment. Subsequently, Bay41-4109-induced aberrant non-capsid polymers are removed by p62-mediated macroautophagy and lysosomal degradation. STUB1 overexpression accelerates Bay41-4109-induced degradation of HBc protein, and thus enhances the effect of Bay41-4109 on inhibiting secretion of HBeAg and HBV virions. When Bay41-4109 are enforced during HBV infection, *de novo* cccDNA formation were also negatively regulated by STUB1 overexpression. Altogether, this study provides novel mechanistic insights into developing more potent and safe HAP-based antiviral treatment.

## Introduction

Chronic hepatitis B virus (HBV) infection results in a significantly elevated risk of developing liver cirrhosis and hepatocellular carcinoma [1]. Currently, the approved therapeutic regimens for HBV include pegylated interferon alpha (IFNα) and nucleos(t)ide analogues (NUCs). IFNα induces the non-cytotoxic intracellular suppression of HBV replication; however, the side effects of IFNα limit its extensive use [1,2]. While NUCs can efficiently suppress HBV DNA titers, they cannot eradicate the virus owing to the persistence of covalently closed circular DNA (cccDNA) that remain in the nuclei of hepatocytes [3]. Thus, new antiviral drugs would be welcomed by the investigators in the field seeking to treat and manage HBV.

The HBV core protein (HBc) is the structural component of the nucleocapsid packaging of the viral genome and DNA polymerase [4]. HBc is viewed as a promising target for antiviral drug development because it participates in multiple steps of the viral life cycle, including RNA packaging, DNA synthesis, viral maturation, and recognition of viral envelope proteins [5]. Drugs known as capsid protein allosteric modulators (CpAMs) have been developed to target HBc. CpAMs can be classified into two types according to their mode of action [6]. Type II CpAMs, represented by sulfamoylbenzamides (SBAs) such as SBA_R01, prevent the assembly of pgRNA-containing nucleocapsids and thereby lead to the production of defective progeny virions with empty capsids [7–9]. Type I CpAMs [10–13], which are represented by heteroaryldihydropyrimidine (HAP) compounds such as Bay41-4109, can mislead HBc into assembling into “aberrant non-capsid polymers”; some type I CpAMs disrupt assembled nucleocapsids by binding to pre-assembled HBc or assembled capsids at the pocket of the dimer–dimer interface, thereby triggering HBc degradation [14–18]. Given that HAP may also induce aberrant polymers of intracellular HBe, treatment with HAPs disrupts the capacity for HBeAg production [17,19]. Although several HAPs are currently undergoing clinical trials, the molecular basis for the degradation of HAP-induced aberrant non-capsid polymers remains unclear.

In the present study, we found that the chaperone-binding E3 ligase STIP1 Homology And U Box-Containing Protein 1 (STUB1) drives Bay41-4109-induced HBc degradation via the macroautophagy-lysosome pathway and that elevation of the STUB1 level enhances the inhibitory effect of Bay41-4109 on the production of both HBeAg and HBV virions.

## Results

### STUB1 mediates Bay41-4109-induced HBc degradation

**Fig 1:**
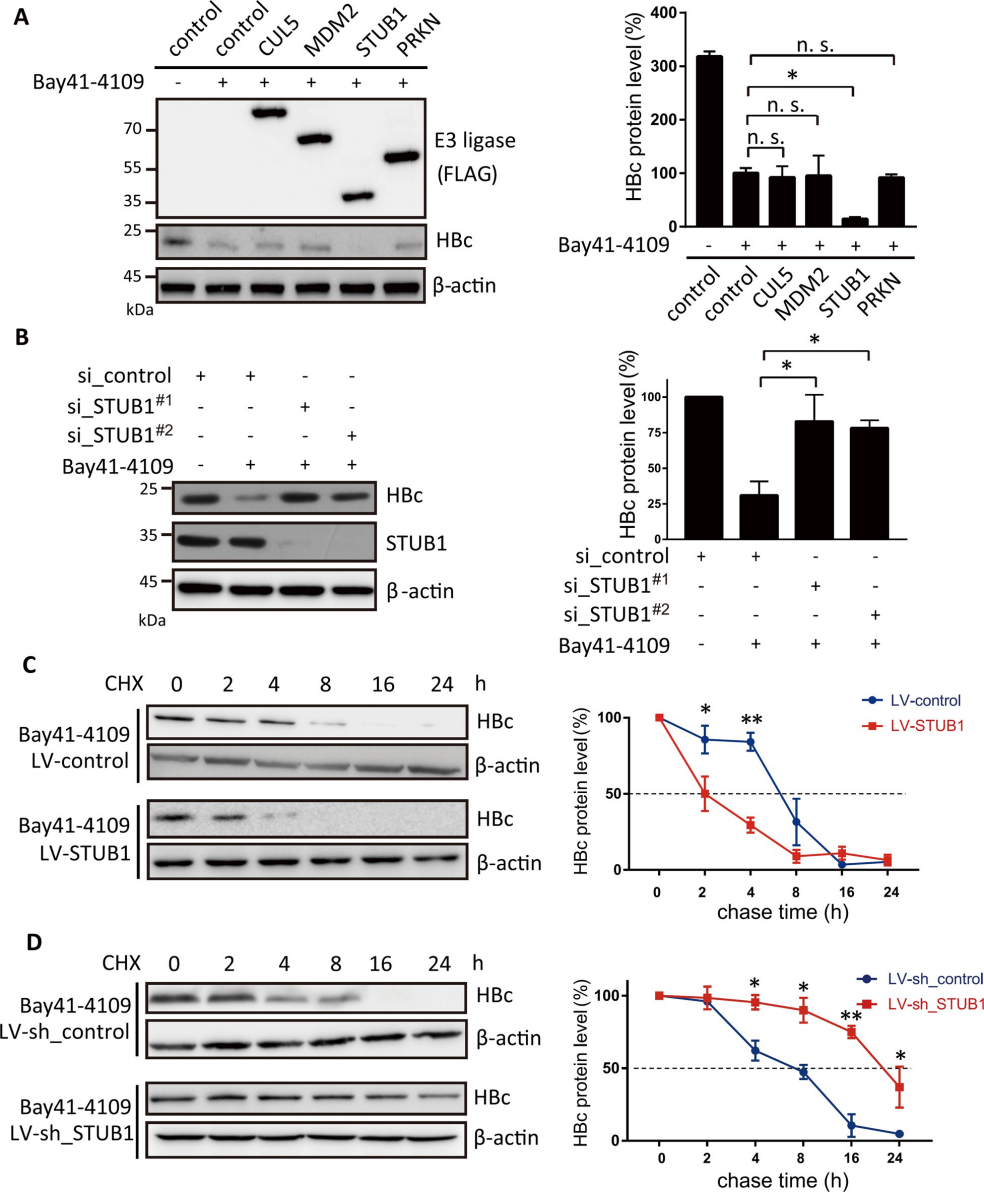
STUB1 promotes the Bay41-4109-induced degradation of HBc. (A, B) HepAD38 cells were transfected with expression vectors coding for E3 ligases including CUL5, MDM2, STUB1, PRKN, or empty vector (A) or two different siRNAs targeting *STUB1* or mock siRNA (B). At 36 hours after transfection, cells were incubated in medium with 1 μM Bay41-4109 or DMSO for 2 d. Cell extracts were then analyzed by western blotting using the indicated antibodies (left panel). The quantification results of three independent immunoblots are shown as relative percentages (HBc/Actin) with mock transfection/transduction samples set to 100% (right panel). The error bars indicate ±SD. Data were analyzed by one-way analysis of variance, followed by Tukey’s comparison test for all groups * indicates *p* < 0.05. n. s. indicates *p* > 0.05. (C, D) HepAD38 cells were transduced with LV-STUB1 or LV-control (C) or with LV-sh_STUB1 or mock LV-sh_control (D). At 36 h after transduction, cells were treated with 1 μM Bay41-4109 and 50 μg/ml CHX for the indicated time. The proteins were detected by western blot using the indicated antibodies (left panel). The quantification results of HBc/actin ratio from two independent immunoblots are shown as relative percentages (right panel). The samples of CHX treatment at 0 h were set to 100%. The error bars indicate ±SD.* indicates *p* < 0.05.**p < 0.01, *p* was calculated by unpaired two-tailed student’s t-test.

HBc is one of the known client proteins of heat shock protein 70 (Hsp70) [20,21]. Some clients of Hsp70 can be degraded by chaperone-binding E3 ubiquitin ligases such as Cullin 5, MDM2, STUB1 and PRKN [22–25]. We hypothesized that one or more of these chaperone-binding ubiquitin E3 ligases may promote HAP-induced HBc degradation. In pursuit of this, we transfected HepAD38 cells [26] (which have a genome containing the 1.1mer HBV genome under the transcriptional control of a tetracycline-responsive promoter (tet-off)) with plasmids for the expression of CUL5, MDM2, STUB1, or PRKN. The overexpression of STUB1, but not the other chaperone-binding E3 ligase, significantly decreased the HBc level in the Bay41-4109-treated cells (Fig 1A).

We next investigated whether STUB1 is required for this observed Bay41-4109-induced reduction of HBc. STUB1 knockdown restored the HBc protein level in Bay41-4109-treated HepAD38 cells (Figs 1B and S1). We also performed a cycloheximide (CHX) chasing experiment in HepAD38 cells with recombinant lentiviruses expressing either STUB1 (LV-STUB1) or shRNA targeting STUB1 (LV-sh_STUB1). Overexpression of STUB1 shortened the half-life of HBc from approximately 6 to 2 h in Bay41-4109-treated cells (Fig 1C). In contrast, the knockdown of STUB1 increased the half-life of HBc from approximately 6 to 22 h (Fig 1D), indicating that STUB1 negatively regulates the stability of HBc in Bay41-4109-treated cells. Of note, manipulation of STUB1 expression only in the cells without Bay41-4109 treatment did not have a significant impact on HBc expression and its degradation (S2 Fig).

### Bay41-4109 induces aberrant non-capsid polymers dependent on STUB1

**Fig 2:**
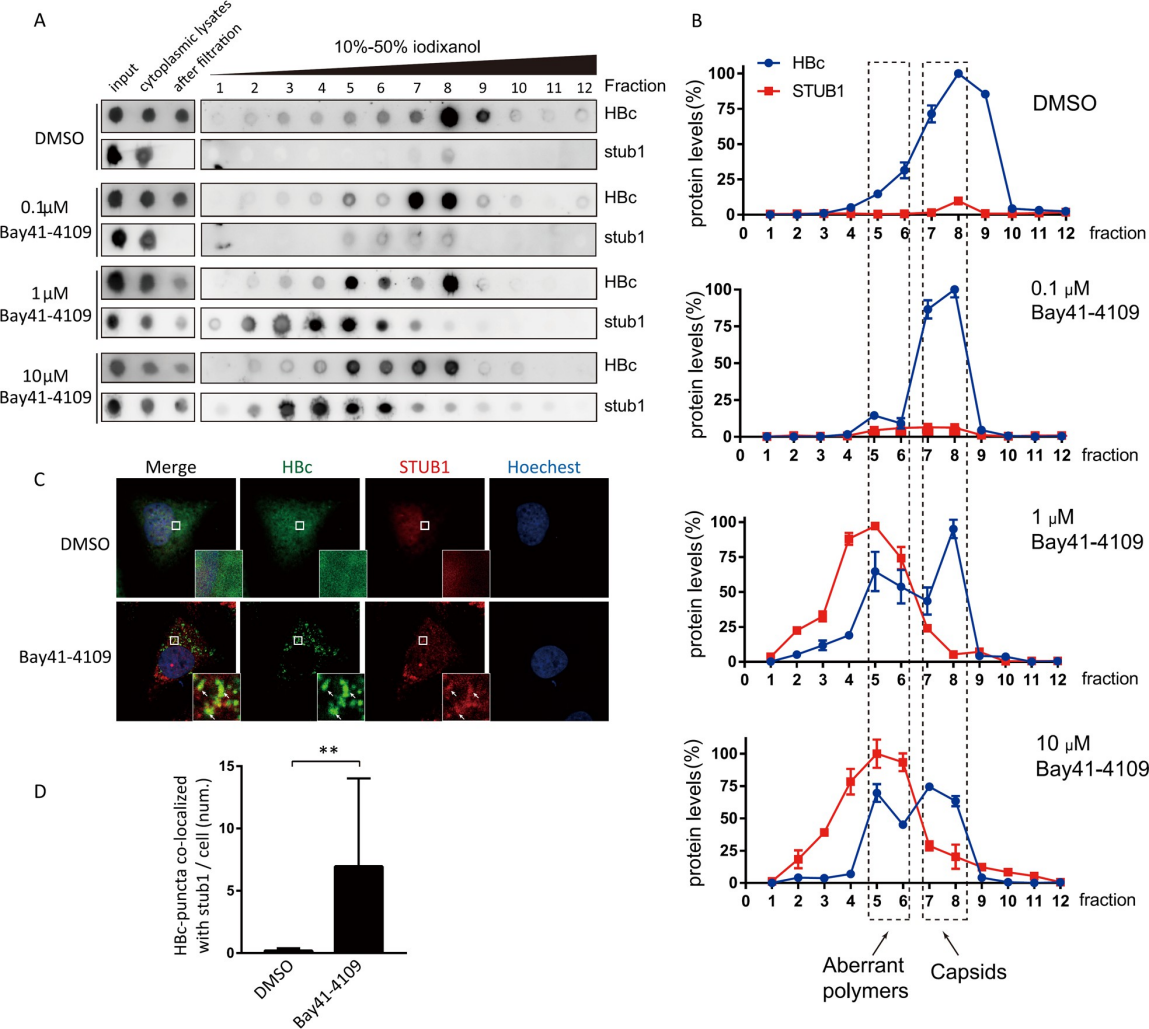
Co-sedimentation and co-localization of STUB1 with Bay41-4109-induced aberrant non-capsid polymers. (A, B) HepAD38 cells were treated with DMSO or increasing concentrations of Bay41-4109 (as indicated) for 36 h. Cytoplasmic lysates from each sample were condensed with a centrifugal filter (100-KDa cutoff) and subjected to iodixanol gradient ultracentrifugation. The levels of the HBc and STUB1 proteins in each fraction were then analyzed with dot blots (A), and were quantified based on two independent experiments by ImageJ (B). (C) HepAD38 cells were treated with 1 μM Bay41-4109 or DMSO as indicated for 48 h. The cells were then immunostained for HBc (green) and stub1 (red). Nuclei were stained with Hochest33342. Areas indicated by white boxes are enlarged. Arrows point to representive co-localized sites. The scale bar is 10 μm. (D) Quantification of the number of HBc puncta co-localized with STUB1 per cell. 100 cells were counted. The error bars indicate ±SD. **p < 0.01, calculated by unpaired two-tailed student’s t-test.

HAPs have been reported to induce the aberrant assembly of viral capsid proteins (as aggregated proteins previously described as “aberrant non-capsid polymers”) [15,17], and the STUB1-based protein quality control system is known to function in degrading misfolded or aggregated proteins [27]. We investigated whether Bay41-4109 induced the formation of these aberrant non-capsid polymers by treating HepAD38 cells with increasing concentrations of Bay41-4109 and performed ultracentrifugation analysis on the cell lysates in iodixanol gradients. In control cells (dimethyl sulfoxide (DMSO)-treated), the amounts of HBc protein peaked in the 8^th^ fraction (Fig 2A and 2B) whereas HBc protein level additionally peaked in the 5^th^ fractions for the cells treated with 1 or 10 μM Bay41-4109. Negative-staining electron microscopy of the 8^th^ fraction from mock-treated cell lysate showed the anticipated icosahedral particles (representing nucleocapsids and/or capsids) (S3 Fig). Fraction 5 from 10 μM Bay41-4109 treated-cell lysates had irregular structures resembling the previously reported aberrant non-capsid polymers. The 5^th^ fraction and the 8^th^ fraction of density gradient samples were also subjected to particle gel assay. When cells were treated with 1 μM or 10 μM Bay41-4109, the bands detected by anti-HBc in the 5^th^ fraction were slightly up-shifted (S4 Fig) as compared to the 8^th^ fraction. This result is similar to the observation of shifted bands of the HAP-induced aberrant polymers in a previous report [28].

Given that STUB1 may interact with the aberrant non-capsid polymers, STUB1 was detected in the polymer-containing fractions of 1 μM and 10 μM Bay41-4109-treated cell lysates (Fig 2A and 2B); only a small amount of STUB1 was detected in the 8^th^ fractions from the control cells, probably because the majority of STUB1 was lost in the filtration before gradient centrifugation provided that aberrant polymers were not induced by Bay41-4109.

We subsequently immunostained Bay41-4109- or mock-treated HepAD38 cells with the STUB1- and HBc-specific antibodies. 1 μM Bay41-4109 showed scattered HBc puncta, which is likely related to formation of aberrant polymers (Fig 2C) [29]. These HBc puncta were partially merged with STUB1 in Bay41-4109 treated cells (Fig 2C) (average 8 puncta in Bay41-4109-treated cells versus 0.3 in control cells) (Fig 2D). These results together demonstrate that Bay41-4109 induces formation of aberrant non-capsid polymers and that STUB1 associates with these polymers.

### STUB1 mediates the lysosomal degradation of Bay41-4109-induced aberrant non-capsid polymers

**Fig 3:**
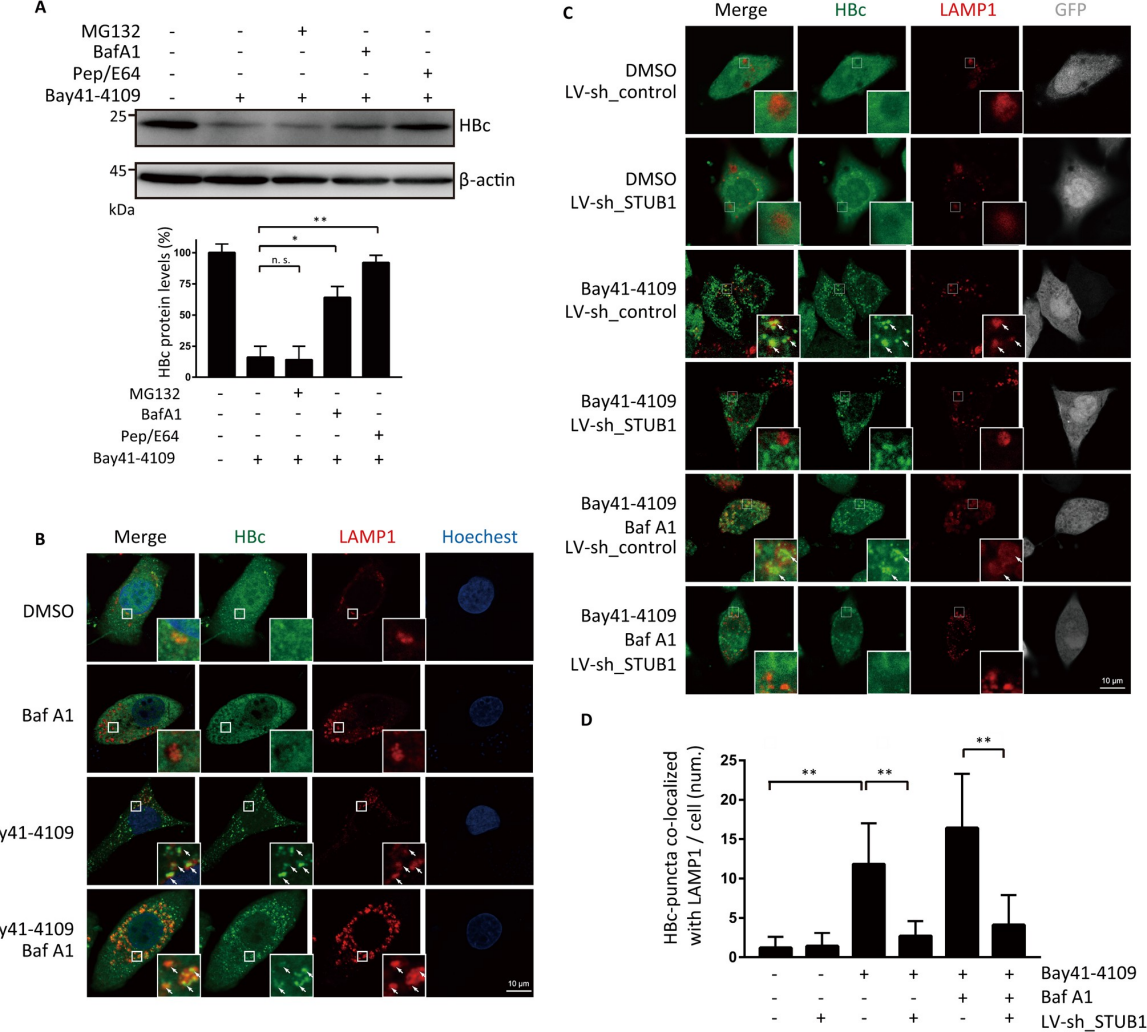
Bay41-4109 induces STUB1-driven lysosomal degradation of HBc. (A) HepAD38 cells were treated with 1 μM Bay41-4109 or DMSO for 24 h, followed by treatment with 5 μM of the proteasome inhibitor MG132, 0.1 mM of lysosome inhibitor BafA1, or 10 mM of lysosome inhibitor Pep/E64 for another 24 h. Cell extracts were then analyzed by western blotting using the indicated antibodies (upper panel). The quantification results of two independent immunoblots are shown as relative percentages (HBc/actin) with the DMSO-treated sample set to 100% (lower panel). The error bars indicate ±SD. **p < 0.01, *p < 0.05, “n. s.” denotes *p* > 0.05. *p* values were calculated by unpaired two-tailed student’s t-test. (B) HepAD38 cells were treated with 1 μM Bay41-4109 or DMSO as indicated for 24 h, followed by treatment with 0.1 mM BafA1 for 12 h. (C) HepAD38 cells transduced with LV-sh_STUB1 or control LV-sh_control were treated with 1 μM Bay41-4109 or DMSO for 24 h. Subsequently, the cells were treated with 0.1 mM BafA1 for 12 h. GFP-positive cells indicate lentivirus transduction (gray). (C, D) The cells were immunostained for HBc (green) and LAMP1 (red). Areas indicated by white boxes are enlarged. Arrows point to typical co-localized sites. Scale bar is 10 μm. (D) Quantification of the number of HBc puncta co-localized with Lamp1 per cell. 100 cells were counted. The error bars indicate ±SD. **p < 0.01, “n. s.” represents *p* > 0.05, *p* values were calculated by unpaired two-tailed student’s t-test.

It is well-documented that STUB1 can promote proteasome- and lysosome-mediated protein degradation [30]. Treatment of cells with inhibitors of lysosomal degradation attenuated Bay41-4109-induced reduction in HBc whereas treatment with a proteasome inhibitor did not disrupt the Bay41-4109-induced reduction in HBc (Figs 3A and S5). These results suggest that Bay41-4109 induces degradation of HBc in a lysosome-dependent manner. Immunostaining showed that Bay41-4109 treatment induced co-localization of HBc with LAMP1 (lysosome marker lysosome-associated membrane protein 1); only background co-localization of HBc and LAMP1 was observed in untreated control cells (Fig 3B). We also investigated the potential role of STUB1 in the lysosomal localization of the Bay41-4109-induced aberrant non-capsid polymers. Knockdown of STUB1 in Bay41-4109-treated cells reduced the extent of HBc’s lysosomal localization (Fig 3C and 3D), suggesting that STUB1’s association with aberrant non-capsid polymers somehow mediates trafficking of the polymers to lysosomes.

### STUB1 drives the p62-mediated macroautophagy of Bay41-4109-induced aberrant non-capsid polymers

**Fig 4:**
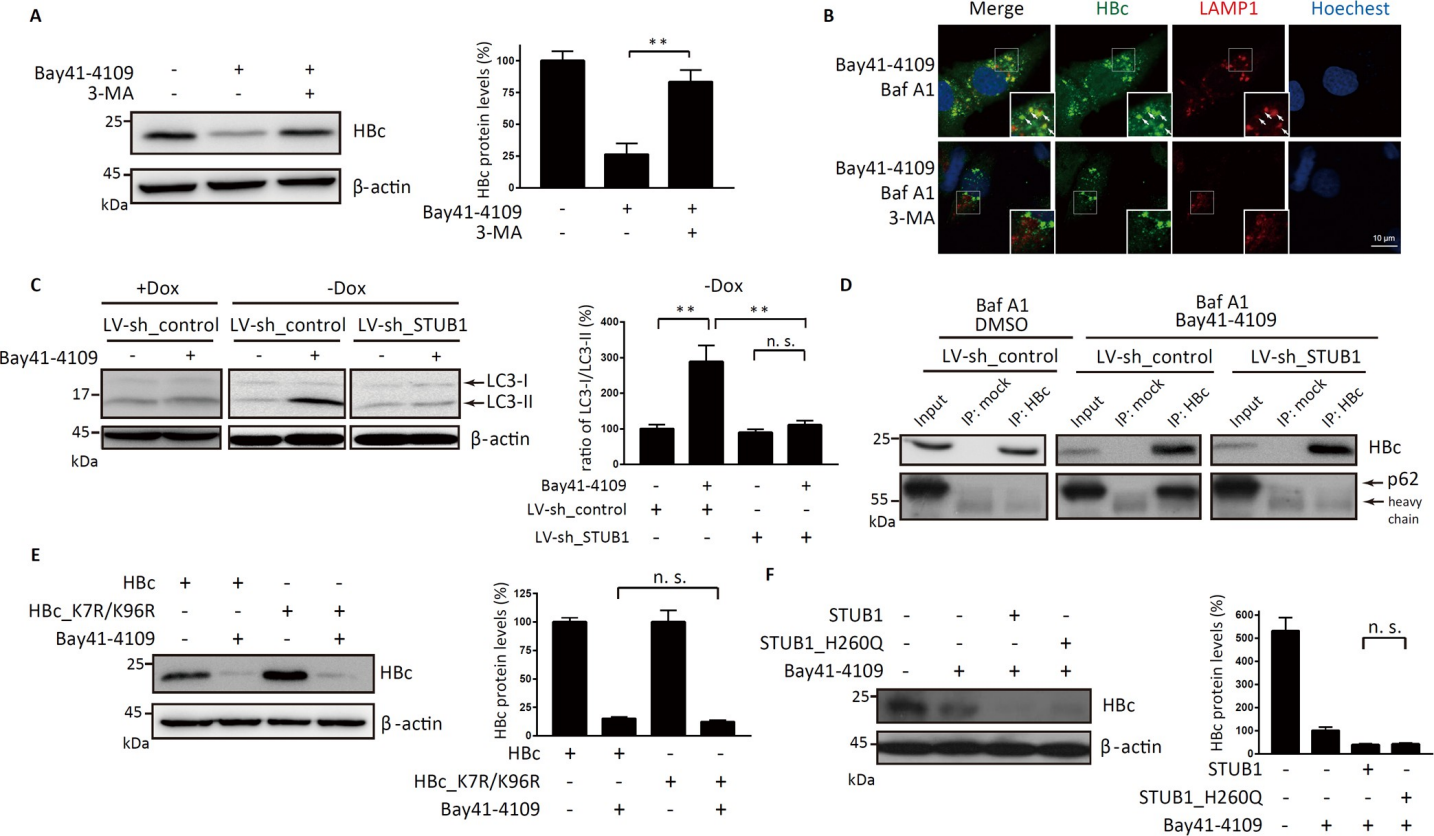
Aberrant non-capsid polymers induced by Bay41-4109 are degraded via STUB1-mediated macroautophagy. (A) HepAD38 cells were treated with 1 μM Bay41 or DMSO for 1 d, followed by treatment with 10 mM 3-MA or DMSO as indicated for 24 h. Proteins were detected by western blot using antibodies recognizing the indicated proteins (left panel). (B) HepAD38 cells were treated with 1 μM Bay41-4109 or DMSO for 24 h followed by treatment with 0.1 mM BafA1 and 10 mM 3-MA (or BafA1 alone) for 12 h. The cells were immunostained for HBc (green) and LAMP1 (red) as indicated. Nuclei were stained with Hochest33342. Areas indicated by white boxes are enlarged. Arrows point to typical co-localized sites. Scale bar is 10 μm. (C) HepAD38 cells were transduced with LV-sh_STUB1 or LV-sh_control, cultured in the the presence or absence of Dox, and treated with 1 μM Bay41-4109 or DMSO for 24 h. (D) HepAD38 cells were transduced with LV-sh_STUB1 or LV-sh_control, treated with 1 μM Bay41-4109 or DMSO for 1 d, and then treated with 0.1 mM BafA1 for 12 h. Cell lysates were immunoprecipitated with HBc antibodies or mock antibodies. Proteins were detected by western blot using antibodies recognizing the indicated proteins. (E) Huh7 cells expressing HBc or lysine-free HBc mutant (HBc_K7R/K96R) were treated with 1 μM Bay41-4109 or DMSO. The proteins were detected by western blot using the indicated antibodies. (F) HepAD38 cells were transfected with STUB1, STUB1_H260Q, or empty vector. At 24 h after transfection, the cells were incubated with or without 1 μM Bay41-4109 for 2 d. The proteins were detected by western blot using the indicated antibodies. (A, B, C, E, F) The right panels show the quantification results of three independent immunoblots as relative percentages (HBc/actin) (A, E, F) or (LC3-I/LC3-II) (C) with the control sample set to 100%. The error bars indicate ±SD. **p < 0.01, *p* value was calculated by unpaired two-tailed student’s t-test.

STUB1 is known to promote the lysosomal degradation of proteins via chaperone-mediated autophagy and p62-mediated macroautophagy [27]. Large cargoes such as protein aggregates and cell organelles are delivered to the lysosomes by p62-mediated macroautophagy [29]. Given the large size of the Bay41-4109-induced aberrant polymers, we incubated Bay41-4109-treated HepAD38 cells with the macroautophagy inhibitor 3-methyladenine (3-MA) and found that 3-MA reversed the Bay41-4109-induced reduction of HBc levels (Fig 4A). Additionally, treatment with 3-MA dramatically decreased the number of HBc-LAMP1 co-localized puncta (from 11 ± 4 to 4 ± 2 puncta per cell) (Fig 4B). Thus, Bay41-4109-induced aberrant non-capsid polymers are trafficked to lysosomes for degradation via macroautophagy.

We further examined the macroautophagy process (including involvement of STUB1) by measuring the LC3-II to LC3-I ratio [31] in lysates of HepAD38 cells. Bay41-4109 promoted the conversion of cytosolic LC3-I to the lipidated, autophagosome-associated LC3-II in the cells expressing HBc, but not in the HBc non-expressed cells (Fig 4C), indicating the aberrant polymer dependent activation of autophagy. Knockdown of STUB1 restored the LC3-II/LC3-I ratio to the level observed in untreated control cells (Fig 4C). These findings support that STUB1 is required for the stimulation of macroautophagy observed in cells with Bay41-4109-induced aberrant polymers.

P62 is a macroautophagy receptor that directs substrate proteins to autophagosome by simultaneously interacting with a substrate protein and with LC3 [31]. We investigated whether Bay41-4109-induced aberrant polymers interact with p62, and assessed whether the interaction may be facilitated by STUB1. Co-immunoprecipitation analysis showed that p62 was detected in the anti-HBc precipitates from Bay41-4109-treated HepAD38 cells but not in the precipitates from DMSO control cells. Of note, owing to the rapid degradation of HBc, the lysosomal inhibitor Baf A1 was included in all samples in these experiments (Fig 4D). No p62 was detected among the anti-HBc precipitates from STUB1 knockdown and Bay41-4109-treated cells (Fig 4D), indicating that STUB1 facilitates the interaction between p62 and Bay41-4109-induced aberrant polymers.

P62-mediated macroautophagy selectively recognizes ubiquitinated substrate proteins although it is known that some non-ubiquitinated proteins can also be degraded via macroautophagy [32]. Our *in vivo* ubiquitination assays showed no obvious ubiquitination of HBc in HepAD38 cells treated with Bay41-4109, even when STUB1 was overexpressed (S6 Fig). Consistently, we found that the level of HBc_K7R/K96R (an HBc mutant missing 2 potential ubiquitin modification sites) [33], was decreased upon treatment of cells with Bay41-4109 (Fig 4E). Moreover, the STUB1 mutant variant STUB1^H260Q^, which lacks E3 ubiquitin ligase activity [23], exhibited the same ability to reduce the HBc protein level as STUB1^wild-type^ (Fig 4F). These results demonstrate that STUB1 drives autophagosome formation for P62-mediated macroautophagy of Bay41-4109-induced aberrant non-capsid polymers. Importantly, this process is independent of STUB1’s E3 ubiquitin ligase activity while our data suggest that STUB1 somehow promotes the interaction between p62 and the aberrant polymers.

### STUB1 mediates the transportation of Bay41-4109-induced aberrant polymers to the perinuclear region

**Fig 5:**
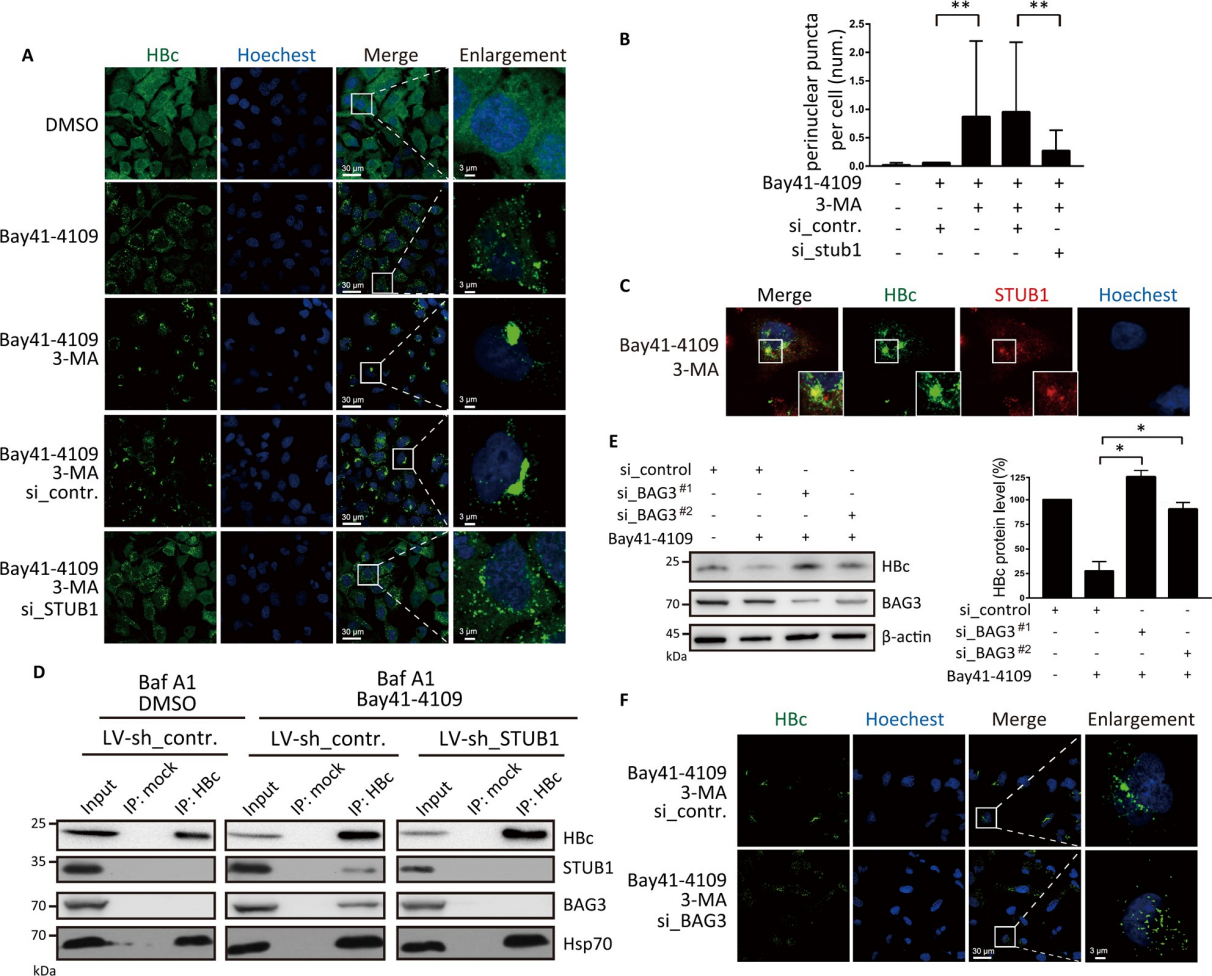
STUB1 mediates transport of Bay41-4109-induced aberrant non-capsid polymers to the perinuclear region. (A) HepAD38 cells were treated with 1 μM Bay41-4109 alone, Bay41-4109 and 10 mM 3-MA, or DMSO for 2 d. For STUB1 silencing, cells were transfected with si_STUB1 or si_control at 36 h before treatment with both Bay41-4109 and 10 mM 3-MA. Cells were immunostained for HBc (green) as indicated. Nuclei were stained with Hochest33342. The “enlargement” column shows enlarged images of the areas indicated by white boxes in the “merge” column. Scale bars are 30 μm in the “merge” column and 3 μm in the “enlargement” column. (B) Numbers of perinuclear HBc puncta (diameter exceeds 3 μM in any direction) per cell. 100 cells were counted. The error bars indicate ±SD. **p < 0.01, *p* value was calculated by unpaired two-tailed student’s t-test. (C) HepAD38 cells were treated with 1 μM Bay41-4109 and 10 mM 3-MA for 2 d. Cells were immunostained for HBc (green) and STUB1 (red) as indicated. Nuclei were stained with Hochest33342. Areas indicated by white boxes are enlarged. Arrows point to typical co-localized sites. Scale bar is 10 μm. (D) HepAD38 cells transduced with LV-sh_STUB1 or LV-sh_control were treated with 1 μM Bay41 or DMSO for 24 h, and all the cells were treated with 0.1 mM BafA1 for 12 h. Cell lysates were immunoprecipitated with HBc antibodies or mock antibodies. Proteins were detected by western blot using antibodies recognizing the respective proteins. (E) HepAD38 cells were transfected with two different siRNAs targeting BAG3 or mock siRNA. At 36 hours after transfection, cells were incubated in medium with 1 μM Bay41-4109 or DMSO for 2 d. Cell extracts were then analyzed by western blotting using the indicated antibodies (left panel). The quantification results of three independent immunoblots are shown as relative percentages (HBc/Actin) with mock transfection/transduction samples set to 100% (right panel). (F) HepAD38 cells were transfected with si_BAG3 or si_control. 36 h after transfection, HepAD38 cells were treated with 1 μM Bay41-4109 and 10 mM 3-MA, or DMSO for 2 d. Cells were immunostained for HBc (green) as indicated. Nuclei were stained with Hochest33342. The “enlargement” column shows enlarged images of the areas indicated by white boxes in the “merge” column. Scale bars are 30 μm in the “merge” column and 3 μm in the “enlargement” column.

A previous study reported that STUB1-bound, aggregated proteins can accumulate in the perinuclear region before being delivered to autophagosomes [27]. We next studied whether a similar process may occur with Bay41-4109-induced aberrant non-capsid polymers. Interestingly, immunostaining with anti-HBc antibody showed that co-treatment of Bay41-4109 with the macroautophagy inhibitor 3-MA led to obvious perinuclear accumulation of HBc puncta whereas no such perinuclear accumulation was observed in cells treated with Bay41-4109 alone. Furthermore, the number of perinuclear puncta was significantly decreased upon STUB1 knockdown (Fig 5A and 5B). These results collectively support that the Bay41-4109-induced aberrant non-capsid polymers are transported to the perinuclear region and that STUB1 is essential for this perinuclear translocation. Moreover, these findings suggest that Bay41-4109-induced aberrant non-capsid polymers are transported from the perinuclear region to lysosomes in a macroautophagy-dependent manner.

We immunostained HepAD38 cells treated with Bay41-4109 and 3-MA against STUB1 and HBc and observed co-localization of STUB1 and HBc in the perinuclear space (Fig 5C). Considering previous studies reporting that the BAG3 protein mediates the transport of STUB1- and Hsp70-bound aggregate proteins to the perinuclear compartment and facilitates the interaction of aggregated proteins with p62[27,29], we next tested whether BAG3, Hsp70 and STUB1 may interact and potentially form a complex with Bay41-4109-induced aberrant non-capsid polymers. Immunoprecipitation using anti-HBc showed that BAG3 and STUB1 were detected in precipitates from Bay41-4109 and Baf A1-treated HepAD38 cells, but not those from DMSO and Baf A1-treated control cells (Fig 5D). No BAG3 was co-immunoprecipited with HBc in STUB1 knockdown cells (Fig 5D). To validate the physiological role of BAG3 in processing Bay41-4109-induced aberrant polymers, we transfected cells with BAG3-targeting siRNA and treated cells with Bay41-4109 or DMSO. Silencing BAG3 restored Bay41-4109-induced degradation and perinuclear accumulation (upon 3-MA co-treatment) of HBc protein (Fig 5E and 5F). Thus, STUB1 facilitates recruitment of BAG3 to the aberrant non-capsid polymers.

Hsp70 binds to the complex of STUB1, BAG3 and Bay41-4109-induced aberrant polymers (Fig 5D). This host chaperone is also required for the degradation of Bay41-4109-induced aberrant polymers as knockdown of Hsp70 abrogated such degradation (S7 Fig). Since Hsp70 binding to unfolded or aggregated proteins can be promoted by a cationic rhodacyanine MKT-077 [34], we assumed that MKT-077 would enhance the degradation of Bay41-4109-induced aberrant polymers. Indeed, MKT-077 further decreased HBc level in the cells exposed to Bay41-4109 (S7 Fig), indicating that enhanced activity of Hsp70 can facilitate Bay41-4109-induced degradation of HBc.

### STUB1 protects cells against toxicity from Bay41-4109-induced aberrant non-capsid polymers

**Fig 6:**
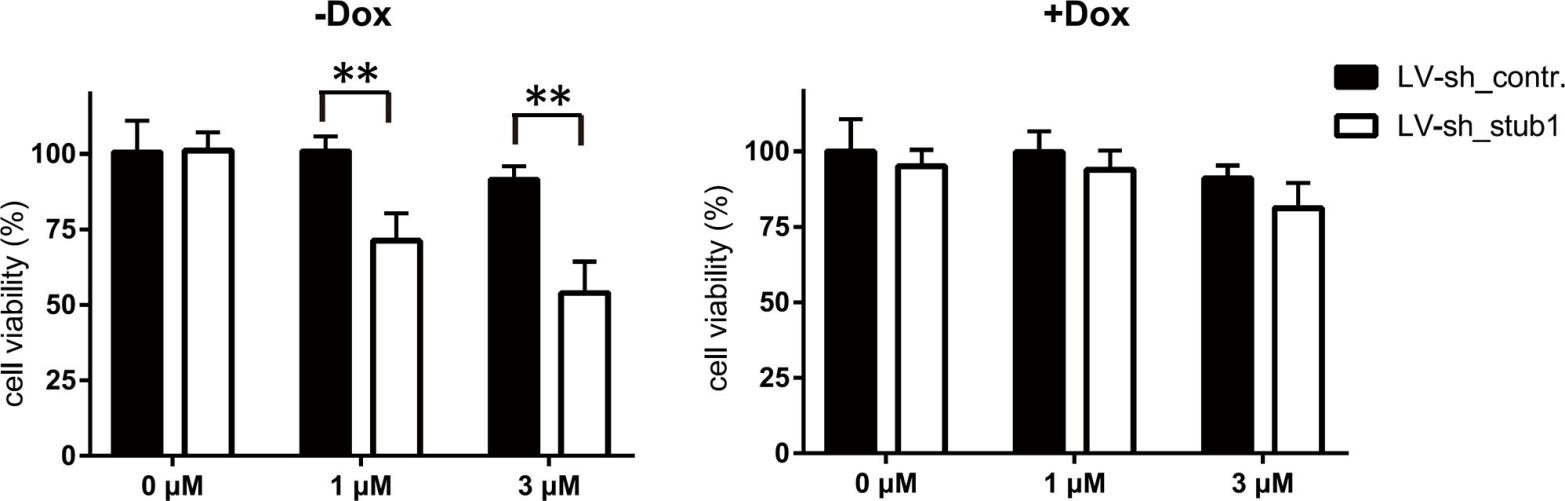
STUB1 protects cells from cytotoxity of Bay41-4109-induced aberrant non-capsid polymers. HepAD38 cells were cultured in the presence (right panel) or absence (left panel) of Dox. Cells were transduced with LV-sh_STUB1 or LV-sh_control. 36 h later, cells were treated with the indicated concentrations of Bay41-4109 for 6 d. A CCK-8 assay was used to quantify cell viability. The error bars indicate ±SD. **p < 0.01, p value was calculated by unpaired two-tailed Student’s t-test.

Many studies have demonstrated that protein aggregates can cause cytotoxicity if they are not removed rapidly [29,35,36]. We investigated whether blocking the degradation of the aberrant non-capsid polymers by knocking down STUB1 could increase cytotoxity. To this end, we knocked down STUB1 by shRNA and treated cells with 1 μM or 3 μM Bay41-4109. These concentrations are 10–35 times lower than the previously reported 50% cytotoxic concentration (CC_50_) of Bay41-4109 [37], but can still induce formation of aberrant non-capsid polymers [32]. STUB1 knockdown significantly decreased the viability of Bay41-4109-treated HepAD38 cells. Furthermore, STUB1 knockdown had no impact on the viability of HepAD38 cells when HBc protein expression was turned off by Dox (Fig 6). These results support that STUB1 reduces the cytotoxicity caused by Bay41-4109-induced aberrant non-capsid polymers, likely by promoting their degradation.

### STUB1 enhanced the inhibitory effect of Bay41-4109 on the production of HBeAg and virions from HepAD38 cells

**Fig 7:**
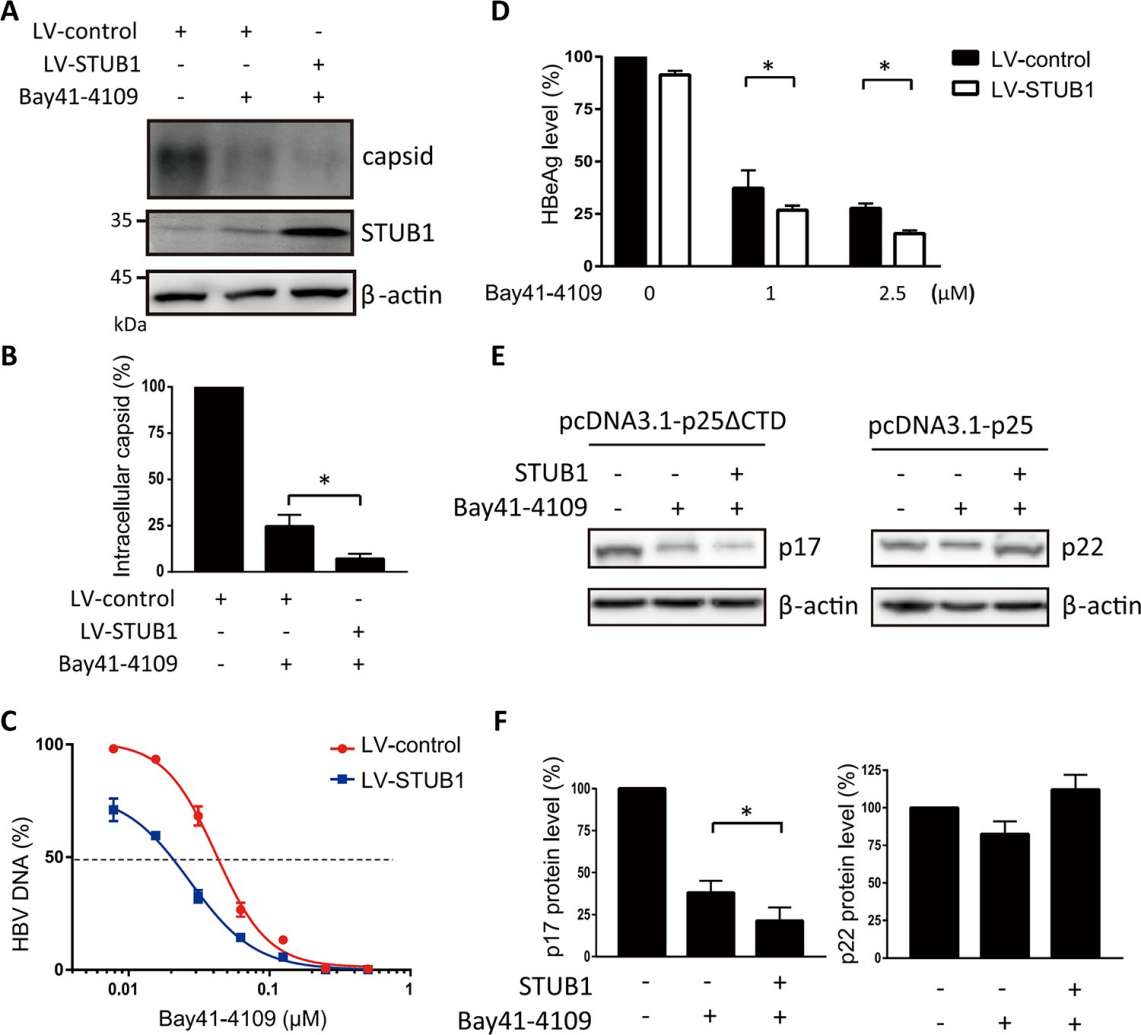
STUB1 inhibits secretion of virions and HBeAg from Bay41-4109-treated HepAD38 cells, and reduces intracellular p17 level in p17-transfected cells. (A, B) HepAD38 cells transduced with LV-STUB1 or LV-control were treated with 1 μM Bay41-4109 or DMSO for 2 d. Capsids precipitated from cell lysates were subjected to agarose gel electrophoresis and western blotting using a rabbit polyclonal anti-HBc antibody (A). Capsid levels of two independent immunoblots were quantified by ImageJ (B). (C, D) HepAD38 cells transduced with LV-STUB1 or LV-control were treated with the indicated concentrations of Bay41-4109 for 6 d. Media were refreshed every 2 d. The number of HBV DNA copies (C) and HBeAg level (D) in media were quantified. The error bars indicate ±SD. **p < 0.01, calculated by unpaired two-tailed student’s t-test. (E, F) HepAD38 cells were transfected with pcDNA3.1-STUB1 or control plasmid, and pcDNA3.1-p25ΔCTD or pcDNA3.1-p25. At 1 d after transfection, cells were incubated in medium with 1 μM Bay41-4109 or DMSO for 2 d. Cell extracts were then analyzed by western blotting using antibodies recognizing myc tag or actin (E). The quantification results of three independent immunoblots are shown as relative percentages (p17/Actin or precore/Actin) with mock-treated samples set to 100% (F).

We reasoned that STUB1-mediated degradation of the aberrant non-capsid polymers upon Bay41-4109 treatment would likely reduce the extent of intracellular capsids, which function in both HBV DNA replication and virion secretion [4]. We therefore assessed whether STUB1 facilitates Bay41-4109’s inhibition with respect to the intracellular capsid and extracellular virion levels. Immunoblot analysis of intracellular capsids that were separated using native agarose electrophorese showed that STUB1 overexpression in Bay41-4109-treated cells reduced the level of intracellular viral capsids by approximately 5 times compared to empty vector control cells (Fig 7B).

Consistently, quantification of HBV DNA derived from extracellular virions demonstrated that overexpression of STUB1 decreased the effective concentration at 50% (EC50) of Bay41-4109 from approximately 42.5 to 23 nM (Fig 7C). No potentiating effects from STUB1 overexpression were observed when HepAD38 cells were treated with a type II CpAM (SBA_R01) (S8 Fig), nor did we observe STUB1-driven transport of HBc to lysosomes in SBA-R01-treated cells (S8 Fig). This is consistent with the known effects of Type II CpAMs for inducing the formation of empty capsids (*i*.*e*., unrelated to the known effects of type I HAPs for inducing formation of aberrant non-capsid polymers).

Moreover, HAP compounds are known to directly reduce HBeAg production because HAP may binds to intracellular HBeAg dimers and induce aberrant polymers [12,17,19]. As STUB1 mediates the degradation of HAP-induced aberrant non-capsid polymers, it is plausible that STUB1 may promote HAP-mediated inhibition of HBeAg secretion. Recent findings demonstrated that in addition to HBeAg, HBc protein can also be secreted by HBV-infected hepatocyte [38–40]. HBc shares 149–159 aa with HBeAg. To distinguish HBeAg from extracellular HBc protein, we used a specific antibody, recognizing the 5^th^ to10^th^ amino acids on the N-terminal residues of HBeAg that has been proven to have no cross-reactivity with HBcAg, to detect HBeAg in cell culture media [41]. The overexpression of STUB1 in Bay41-4109-treated HepAD38 cells (by LV-STUB1 transduction) significantly lowered the level of HBeAg in culture supernatants (Fig 7D). In contrast, the extracellular level of HBsAg—which is not involved in HAP-induced aberrant non-capsid polymers—was not affected by STUB1 overexpression in Bay41-4109-treated cells (S9 Fig). It has been demonstrated that HAP reduces the level of intracellular HBe (p17) [19] or precore protein (p22), i.e. the precursor of HBe [17]. We next evaluated whether STUB1 affects the level of intracellular precore protein or HBe protein. We constructed precore and p17 expression plasmids by inserting the signal peptide-included and c-terminal Myc-tagged p25 ORF sequence or p25ΔCTD into the pcDNA3.1, respectively. As previsously reported, transfection of signal peptide-included p25 and p25ΔCTD leads to expression of the signal peptide-deleted precore protein p22 and p17, respectively [38,42]. p17 level was significantly reduced by Bay41-4109 treatment and further lowered upon STUB1 overexpression (Fig 7E and 7F); the level of precore protein was slightly reduced by Bay41-4109 treatment, but was not further affected by STUB1 overexpression (Fig 7E and 7F). These results supported that Bay41-4109 reduces the level of intracellular p17 by a STUB1-mediated degradation, which may lead to a lower level of secreted HBeAg.

### STUB1 overexpression enhances the inhibitory effects of Bay41-4109 against secretion of HBV virions and HBeAg, and cccDNA formation in HBV-infected HepG2-NTCP cells

**Fig 8:**
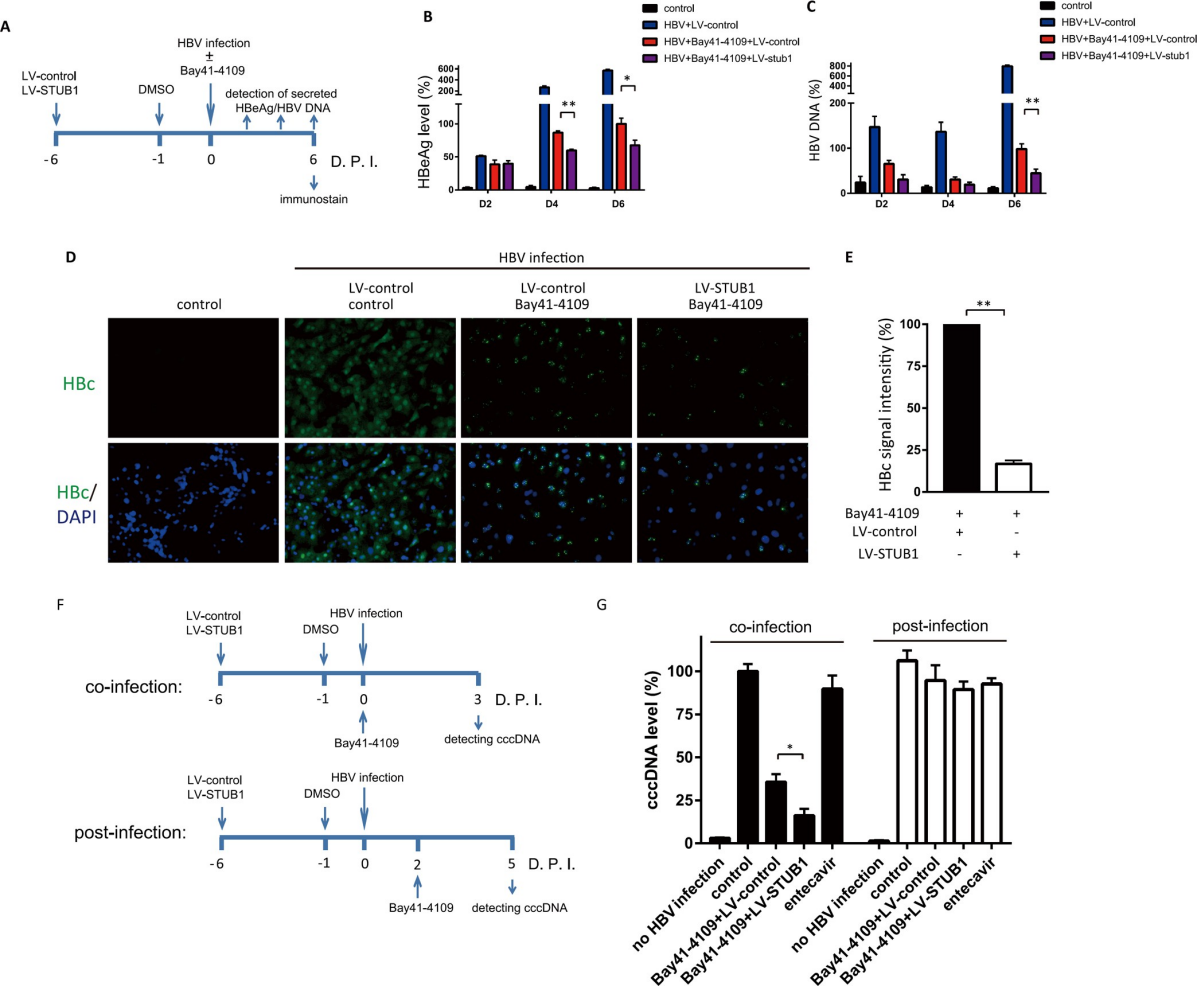
The effect of STUB1 overexpression in Bay41-4109-treated HBV-infected HepG2-NTCP cells. (A) Schematic diagram of the experimental procedure for HepG2-NTCP cells in Fig 8B–8D. (B, C, D) HepG2-NTCP cells transduced with LV-STUB1 or LV-control were infected with HBV at MOI of 500 genome equivalents in the presence of 2% DMSO. Bay41-4109 (1 μM) or DMSO was added during HBV infection. The HBeAg level (B) and HBV DNA copies (C) in media were quantified at 2, 4, and 6 d after HBV infection. The error bars indicate ±SD.*p < 0.05.**p < 0.01, *p* value was calculated by unpaired two-tailed student’s t-test. (D, E) Cells were immunostained for HBc (green) as indicated. Nuclei were stained with Hochest33342 (D) The fluorescence signal intensity was quantified by ImageJ (E). *p < 0.05, *p* value was calculated by unpaired two-tailed student’s t-test. (F) Schematic diagram of the experimental procedure of adding Bay41-4109 during (co-infection) or post HBV infection (post-infection) in LV-control- or LV-STUB1-transduced HepG2-NTCP cells. (G) Cells were treated with Bay41-4109 during (co-infection) or post HBV infection as descripted in Fig 8G. cccDNA were isolated by hirt extraction, and their levels were quantified by qPCR.

We next examined the influence of STUB1 on the inhibitory effect of Bay41-4109 in the context of HBV infection. We initially transduced HepG2-NTCP cells with LV-STUB1 or LV-control and infected these cells with HBV virions 6 days later (day 0). These cells were treated with Bay41-4109 over a 6-day time course, and the supernatant was collected every other day for the quantification of secrected HBeAg and HBV DNA levels (Fig 8A). By 6 days after HBV infection, STUB1 overexpression in Bay41-4109-treated cells lead to a 50% decrease in extracellular HBeAg and a 60% decrease in extracellular HBV DNA (Fig 8B and 8C). Furthermore, we did not observed a decrease in the viability of LV-STUB1-transduced cells in HBV infection assay, so the reduction of HBeAg and virions were not related to the cytotoxicity caused by long-term overexpression of STUB1 (S10 Fig). Immunostaining against HBc at 6 d after HBV infection showed that more than 85% cells were infected by HBV (Fig 8D). Treatment with Bay41-4109 reduced the fluorescence signal intensity of the HBc protein, and induced HBc puncta. STUB1 overexpression in Bay41-4109-treated cells further decreased Bay41-4109-induced puncta of HBc protein (Fig 8D and 8E); consistently, STUB1 overexpression reduce the total HBc protein level in Bay41-4109-treated, HBV-infected cells as detected by immunoblot (S11 Fig). When STUB1 was knocked down by LV-sh_STUB1, the fluorescence intensity of Bay41-4109-induced HBc puncta increased (S12 Fig). Altogether, these findings are consistent with our earlier results from the HepAD38 cells and support that STUB1 can promote Bay41-4109’s anti-viral efficacy.

It has been demonstrated that HAP compound acts on capsid of incoming virus particles and inhibits the infectivity in *de novo* HBV infection and cccDNA synthesis [16,28]. After establishment of cccDNA, HAP can also mislead progeny nucleocapsid assembly. It is very interesting to know whether STUB1 enhances this early effect of HAP on the procedure before *de novo* cccDNA synthesis. We transduced HepG2-NTCP cells with LV-STUB1 or LV-control. 6 days later, we treated the cells with Bay41-4109 at the same time as HBV infection, and quantified cccDNA level at day 3 post HBV infection (Fig 8F). Interestingly, qPCR results demonstrated that STUB1 overexpression further decreased cccDNA levels in Bay41-4109-treated cells (Fig 8G).

It is known that cccDNA level reaches the maximum at 2 day post HBV infection in HepG2-NTCP cells [16]. It is interesting to unveal whether Bay41-4109 could reduce cccDNA level after cccDNA formation has been established, and whether STUB1 can enhance Bay41-4109-induced cccDNA degradation, if any. We treated HBV-infected, LV-STUB1- or LV-control-transduced HepG2-NTCP cells with Bay41-4109 at 2 days after HBV infection, and harvest cells at day 5 post HBV infection (Fig 8F). Intriguingly, cccDNA level was not affected when Bay41-4109 was enforced after HBV infection (Fig 8G). STUB1 overexpression caused no reduction of cccDNA level. Altogether, Bay41-4109 and STUB1 overexpression reduced early *de novo* synthesis of cccDNA, but not induced cccDNA level reduction after HBV infection has established.

### Elevating the STUB1 level enhances the inhibitory effects of Bay41-4109 against production of HBV virions and HBeAg in HBV transgenic mice

**Fig 9:**
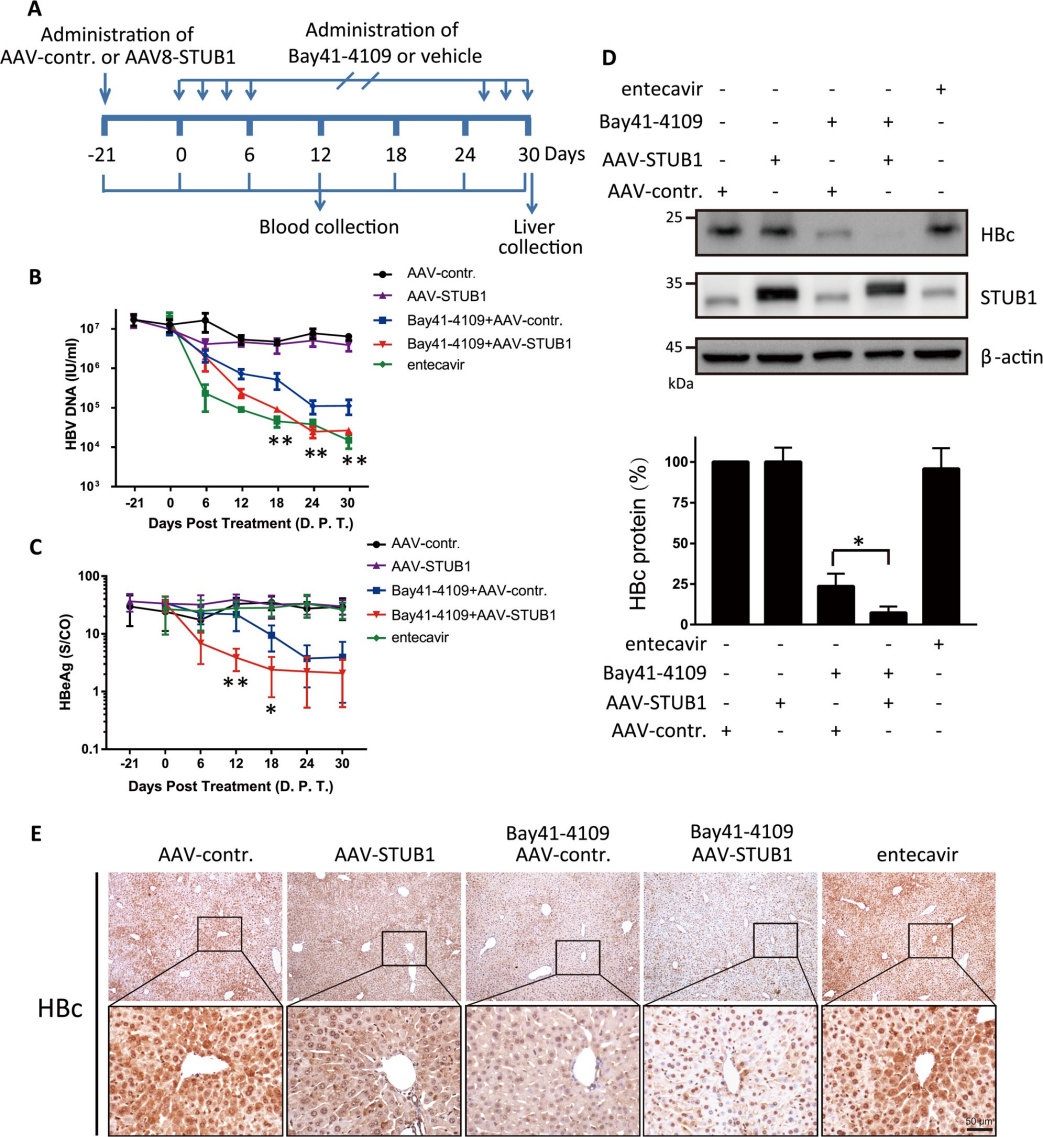
STUB1 enhances Bay41-4109’s inhibitory effect in HBV transgenic mice. (A) Schematic diagram of the procedure for treating HBV transgenic mice. (B, C) Levels of HBV DNA (B) and HBeAg (C) in serum were quantified every 6 d. S/CO is the signal-to-cutoff ratio. The error bars indicate ±SD. Bay41-4109-treated AAV-STUB1 group and Bay41-4109-treated AAV-control group were analyzed by unpaired two-tailed student’s t-test. *p < 0.05.**p < 0.01. (D) The indicated proteins in the livers of each group were detected with immunoblotting (upper panel). The levels of HBc were quantified in three mice of each group (lower panel). (E) The levels of intracellular HBc in the livers were detected by IHC analysis. Scale bar, 50 μm.

HBV transgenic mice can productively replicate HBV in the liver and secrete viral particles and antigens in blood [43]. We evaluated whether modulating the STUB1 level *in vivo* may affect the inhibitory effects of Bay41-4109 on serum HBV DNA levels, on serum HBeAg levels, and/or on intracellular HBc protein levels in liver cells. Recombinant adeno-associated virus 8 (AAV8) was previously demonstrated to achieve sustained gene expression in hepatocytes [44]. Mice were intravenously injected (tail vein) with recombinant adeno-associated virus 8 expressing STUB1 (AAV-STUB1) or a control adeno-associated virus (AAV-control), and were subsequently treated with Bay41-4109 (as summarized in Fig 9A); entecavir was orally administrated once daily as a control. Serum HBV DNA and HBeAg were monitored every 6 d after the first administration of Bay41-4109. Quantification of HBV DNA in mouse serum (Fig 9B) showed that Bay41-4109 treatment led to a sustained decrease in HBV DNA, with a maximum inhibition of 1.8 log10 copies/ml reached at the end of the observation period. The overexpression of STUB1 in mice administered with Bay41-4109 resulted in an additional inhibition of serum HBV DNA (to approximately 1 log10 starting from day 18). Notably, this synergistic inhibition was comparable to the efficacy of entecavir on serum HBV DNA from day 24.

We also observed that compared to the Bay41-4109-treated AAV-control group mice, serum HBeAg levels decreased more rapidly in the Bay41-4109-treated AAV-STUB1 group mice (Fig 9C). Similar to our earlier results with HepAD38 cells, overexpression of STUB1 in Bay41-4109-treated mice showed no further reduction in serum HBsAg, as compared to the Bay41-4109-treated AAV-control group mice (S13 Fig).

At day 30, the mice were sacrificed. The expression level of the HBc protein in liver tissue was analyzed by western blot analysis and immunohistochemistry (IHC) staining. Treatment with Bay41-4109 reduced the intrahepatic HBc protein level, and overexpression of STUB1 in Bay41-4109-treated mice resulted in a significant additional reduction in the levels of intrahepatic HBc protein (Fig 9D and 9E). Notably, compared to the control group (AAV-control), the AAV-STUB1 group had no obvious reduction in the levels of intracellular HBc protein, serum HBV DNA titer, or serum HBeAg. Taken together, these findings support that elevating the level of STUB1 in Bay41-4109-administred mice further decreases the serum viral load and the HBeAg level.

## Discussion

Recent progress has seen the development of CpAMs as clinically or pre-clinically promising anti-HBV candidate drugs. HAPs are Type I CpAMs that exert their effects by “misleading” HBV capsids into assembly of aberrant non-capsid polymers and ultimately inducing HBc degradation [12,45]. In addition to suppressing HBV DNA replication, HAPs have been shown to reduce HBV cccDNA biogenesis and to directly inhibit HBeAg production. Because HAPs reduce the number of functional capsids (which transport viral rcDNA to the nucleus for cccDNA formation), HAPs are capable of decreasing the HBV cccDNA pool during *de novo* infections [16]. Moreover, HAPs directly inhibit HBeAg production [17]. In the mechanism underlying this inhibition, HAP may binds to intracellular HBe (p17) dimers andinduce aberrant polymer [17,19].

**Fig 10:**
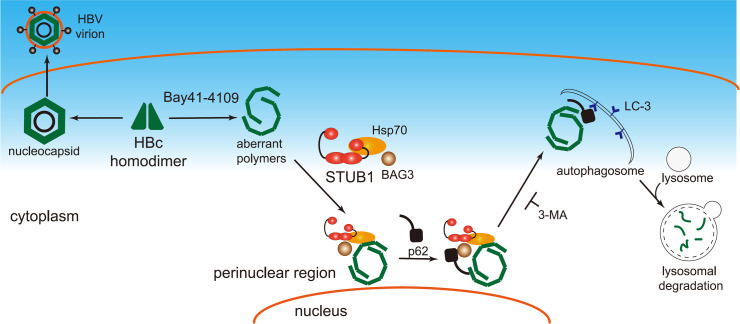
Working model. HAP-induced aberrant non-capsid polymers form complexes with STUB1, Hsp70 and BAG3, and are transported to the perinuclear compartment. Subsequently, macroautophagy receptor p62 binds to the complexes (via interaction with BAG3) and directs aberrant non-capsid polymers to form macroautophagosomes, which are ultimately fused with lysosomes.

In the present study, we demonstrated that Bay41-4109-induced aberrant non-capsid polymers are removed by STUB1-mediated macroautophagy–lysosomal degradation. Our data support the working model for STUB1-driven degradation of HAP-induced aberrant non-capsid polymers (Fig 10). First, HAP-induced aberrant non-capsid polymers form complexes with STUB1, Hsp70 and BAG3, and are transported to the perinuclear compartment. Subsequently, macroautophagy receptor p62 binds to the complexes via an interaction with BAG3 to specify the formation of macroautophagosomes, which ultimately fuse with lysosomes. STUB1 facilitates recruitment of BAG3 thus promoting the degradation of aberrant non-capsid polymers via the p62-mediated macroautophagy–lysosome pathway.

Based on the overexpression and knockdown of STUB1, we found that STUB1 is required for the HAP-induced degradation of HBc. STUB1 co-immunoprecipitated and co-sedimented with HAP-induced aberrant non-capsid polymers; it did not obviously interact with HBc dimers or capsids in cells lacking HAP treatment, supporting that HAP facilitates the interaction between STUB1 and HBc. These results explain the previously reported findings that HBc is very stable when no HAP is present and the half-life of HBc is drastically reduced upon HAP treatment [12].

Previous studies have shown that STUB1 can drive protein degradation via a BAG1-dependent proteasome manner; and STUB1’s function in catalyzing ubiquitin modification of protein substrates has been established [30]. Notably, a previous study showed that treatment with a proteasomal inhibitor (lactacystin) could block a HAP-induced reduction of the HBc protein level [12]. However, we did not observe an increase in the HBc level upon treatment of Bay41-4109-treated HepAD38 cells with proteasomal inhibitors (lactacystin or MG132). STUB1 is also known to facilitate lysosomal degradation of proteins by chaperone-mediated autophagy[46] or p62-mediated macroautophagy [30,47]. In chaperone-mediated autophagy, single proteins that have KFERQ-like motifs can bind to the receptor LAMP2A, after which they are transported to intact lysosomes. In contrast, in p62-mediated macroautophagy, large cargoes such as aggregated proteins or cell organelles can be transported to lysosomes. P62-mediated macroautophagy involves the formation of isolating membranes which engulf proteins in autophagosomes and subsequently fuse with lysosomes [29]. In the present study, by inhibiting lysosomal proteolysis and p62-mediated macroautophagy, we provide evidence that HAP-induced aberrant non-capsid polymers are removed via a p62-mediated macroautophagy–lysosomal pathway. Importantly, our results establish that ubiquitination of HBc is not required for HAP-induced degradation and STUB1 promotes the recruitment of BAG3 to HAP-induced aberrant non-capsid polymers. BAG3 is known to transport cargos to the perinuclear region and to link cargoes to macroautophagy via interaction with the autophagy receptor p62 [27,31].

In Bay41-4109-treated HepAD38 cells, HBV-infected HepG2-NTCP cells and HBV transgenic mice, we demonstrated that STUB1 overexpression leads to a further decrease in the levels of extracellular HBV virions and HBeAg. Reduced HBeAg production may improve therapeutic outcomes by inhibiting the HBeAg-mediated negative modulation of innate and adaptive immune responses to HBV infection. Notably, intensifying the suppression of virion production has been considered as an attractive strategy for eradicating HBV chronic infection. The highly efficient inhibition of viral replication can cause the rate of infection of new hepatocytes to be lower than the rate of infected hepatocyte loss (through natural death or immune clearance). This situation would result in a progressive reduction in the number of infected hepatocytes, potentially extending until no infected hepatocytes remain. Owing to the metabolic instability of HAPs, a co-dosing strategy that combines HAPs with P450 inhibitor drugs has been applied to enhance inhibitory effects in clinical trials, for example with the HAP compound GSL4 [13,48]. Perhaps use of some protein-turnover-related agent (or gene therapy) which can elevate STUB1 levels can be exploited alongside type I CpAMs to achieve lower serum HBV DNA titers and higher anti-HBeAg seroconversion rates. Taken together, our findings pave the way towards the clinical application of type I CpAMs as a potentially curative regimen (or a component of a combination treatment) for eradicating HBV from hepatocytes of chronic infection patients.

## Materials and methods

### Ethics statement

All work performed with animals was in accordance with and approved by the Institutional Animal Care and Use Committee at Fujian Medical University.

### Plasmid construct

STUB1 (Accession: NM_005861), CUL5 (Accession: NM_003478), MDM2 (Accession: NM_001145337), and PRKN (NM_004562) cDNA sequences with FLAG tags fused at the C-terminals were amplified from plasmids purchased from Youbio Co. (Changsha, China). HBc were amplified from HBV DNA of strain 56 (accession: AF100309) kept in our laboratory. All PCR-generated products were inserted into the plasmid pcDNA3.1/(+). FLAG-fused STUB1 and myc-fused HBc were additionally inserted into the plasmids pCDH-CMV-MCS-EF1-T2A-puro and pCDH-CMV-MCS-EF1-T2A-GFP. FLAG-tagged STUB1 was inserted into the plasmid pShuttle. The constructs are detailed in Table 1.

**Table 1:** The constructs information in the study.

Genes	Vectors	Restriction sites	Forward primers	revese primers
CUL5	pcDNA3.1/(+)	EcoRI, NotI	GCGAATTCGCCACCATGGCGACGTCTAATCTGTT	TTGCGGCCGCTTACTTATCGTCGTCATCCTTGTAATCTGCCATATATATGAAA
MDM2	pcDNA3.1/(+)	EcoRI, NotI	GCGAATTCGCCACCATGATAGTGTTTGTCAGGT	TTGCGGCCGCCTACTTATCGTCGTCATCCTTGTAATCCACGTCGAACCAGTGG
PRKN	pcDNA3.1/(+)	EcoRI, NotI	GCGAATTCGCCACCATGATAGTGTTTGTCAGGT	ATGCGGCCGCCTACTTATCGTCGTCATCCTTGTAATCGGGGAAATAAGTTAGC
STUB1	pcDNA3.1/(+)	HindIII, BamH I	TTAAGCTTGCCACCATGAAGGGCAAGGAGGAGA	GTGGATCCTCACTTGTCGTCATCGTCTTTGTAGTCGTAGTCCTCCACCCAGCC
STUB1	pCDH-CMV-MCS-EF1-T2A-puro	HindIII, BamH I	TGGCTAGCGCCACCATGAAGGGCAAGGAGGAG	GAGCGGCCGCCTTGTCGTCATCGTCTTTGTAGTCGTAGTCCTCCACCCAGCC
HBc	pcDNA3.1/(+)	HindIII, BamH I	TTAAGCTTGCCACCATGGACATCGACCCTTATAAAG	GTGGATCCCTACAGATCCTCTTCTGAGATGAGTTTTTGTTCACATTGAGGTTCCCGAGATT
HBc	pCDH-CMV-MCS-EF1-T2A-puro	NheI, BamH I	TGGCTAGCGCCACCATGGACATCGACCCTTAT	GAGCGGCCGCCAGATCCTCTTCTGAGATGAGTTTTTGTTCACATTGAGGTTCCCGAGA

HBc_K7R/K96R, p25, p25ΔCTD and STUB1-H260Q were chemically synthesized by General Biosystems (Anhui, China).

### Cell culture and transfection

The human hepatoma cell lines HepG2 (HB-8065; American Type Culture Collection [ATCC], Manassas, VA) and Huh7 (JCRB0403; Japan) were cultured in Dulbecco’s modified Eagle medium (DMEM; Thermo Fisher Scientific, Waltham, MA, USA), supplemented with 10% fetal bovine serum (FBS) and maintained in a humidified atmosphere containing 5% CO2 at 37°C. HepAD38 cells were purchased from the Shanghai Second Military Medical University and maintained in DMEM with 10% FBS, 1% sodium pyruvate, 3 μg/ml Doxycycline, and 400 μg/ml G418 (Catalog no. 345810, Sigma). When Doxycycline was removed from the culture medium, the HepAD38 cells secreted HBV virions. To detect the HBc protein level, determine the LC3I/LC3II ratio, and immunostain HBc, LAMP1, and STUB1, the following chemicals were used: 1 μM Bay41-4109 (MedChemExpress, HY 100029), 10 mM 3-MA (MedChemExpress, HY-19312), 2 μM SBA_R01 (synthesized by WuXi AppTec), 0.1 mM BafA1 (MedChemExpress, HY-100558), 10 mM pepstatin A (selleck, s7381) and E64 (MedChemExpress, HY-15282), and 5 μM MG132 (Beyotime, s1748). Transient transfections were performed using the Lipofectamine 3000 transfection reagent (Invitrogen) according to the manufacturer’s instructions. For STUB1 silencing, siRNAs chemically synthesized by Shanghai GenePharma Co. (Shanghai, China) was used. Scramble siRNA (NC-siRNA) was used as a negative control. For Bay41-4109 treatment, 1 μM Bay41-4109 or DMSO was added to the cell culture medium at 36 h post-transfection.

The siRNA-targeting sequences are listed in Table 2.

**Table 2:** The siRNA sequences used in the study.

Genes	Target sequence 1	Target sequence 2
STUB1	CCAAGCACGACAAGUACAUTT	GGCAAUCGUCUGUUCGUGGGCCGAAA
CUL5	CTGGAGGACTTGATACCGGAA	CCAGCTGATTCAGTTATTATA
MDM2	GCUUCGGAACAAGAGACUC	AAGCCAUUGCUUUUGAAGUUA
Parkin	CCAACUCCCUGAUUAAAGATT	UUCGCAGGUGACUUUCCUCUGGUCA
BAG3	AACAGGUGCAGUUUCUCGAUGGGUC	AAGGUUCAGACCAUCUUGGAA
Hsp70	GGACATCAGCCAGAACAAG	CTGGCCTTTCCAGGTGATC

### Generation and usage of recombinant adenoviruses and lentiviruses

The generation of HBc- or STUB1-encoded lentiviruses or adenoviruses and the infection of HepAD38 or Huh7 cells were performed as we previously described [49]. Stable HBc- and HBc-KR-expressed cell lines were constructed by infecting Huh7 cells with HBc or HBc-K7R/K96R-encoding lentiviruses and screening with 1 μg/ml puromycin for one week.For silencing, HepG2 and HepAD38 cells were seeded in six-well plates at a density of 5 × 10^5^ cells per well and infected with recombinant lentiviruses encoding GFP (LV-contr.) or STUB1 (LV-STUB1). Cells were harvested at 48 h post-infection for co-immunoprecipitations. Lentivirus carrying shRNA targeting STUB1 were purchased from Genepharma. The shRNA sequence for STUB1 was 5’-TGCCGCCACTATCTGTGTAAT-3’, which targets the 3’-UTR of STUB1 mRNA. For rescuing, STUB1-deficient cells were transfected with pcDNA3.1-STUB1, whose expression is resistant to STUB1-specific shRNA.

### Western blot analysis

Cell lysates and western blotting were performed as described previously [49]. The specific antibodies used in this study included anti-HBc (1:1000, Abcam, ab8639), anti-β-actin (1:2000, Sigma, A2228), anti-FLAG (1:1000, cell signaling, #2368), anti-myc (1:1000, cell signaling, #2276), anti-HSP70 (1:1000, cell signaling, #4876), anti-STUB1 (1:5000, abcam, ab134064), biotin-conjugated anti-HBc (for detecting immunoprecipitation), anti-LC3A/B (1:500, cell signaling, #12741), anti-p62 (1:1000, abcam, ab56416), and anti-bag3 (1:1000, abcam, ab92309).

### Determination of HBc half-life

HepAD38 cells were infected with LV-STUB1, LV-contr., LV-sh_STUB1, or LV-sh_control for 36 h and then treated with 1 μM Bay41-4109 for 36 h or DMSO as a control. Subsequently, the cells were incubated with 50 μg/ml CHX (Cell Signaling Technology) for the indicated time. After washing with pre-chilled PBS, the cells were lysed with RIPA buffer containing a protease inhibitor cocktail (Roche Diagnostics). Proteins were separated by SDS-PAGE and western blot.

### Cellular immunofluorescence staining

HepAD38 cells were infected with LV-sh_STUB1 or LV-contr. for 24 h and then treated with 1 μM Bay41-4109 for 48 h, 0.1 μM Baf A1 for 12 h, 10 mM 3-MA for 12 h, or a combination of them as indicated. The cells were washed with PBS, fixed in 4% paraformaldehyde, and permeabilized with 0.1% Triton X-100 (Bio-Rad). The cells were then stained with anti-HBc (1:1000, Dako, B0586), anti-p62 (1:50, abcam, ab256416), anti-LAMP1 (1:40, abcam, ab25630), or anti-Hsp70 (1:200, abcam, ab2787) followed by incubation with AlexaFluor 594-, AlexaFluor 488-, or DyLight 405-conjugated secondary antibodies (cell signaling, #8890 or #4408; abbkine, A23120) for the co-localization of HBc with LAMP1. For the double staining of HBc and STUB1, the primary antibodies anti-HBc (1:100, abcam, ab8637) and anti-STUB1 (1:200, abcam, ab2917) were used. Fluorescent images were acquired using a Leica TCS SP8 confocal microscope.

### Co-immunoprecipitation assays

HepAD38 cells treated as indicated were lysed, and the soluble proteins were pre-cleared with 100 μl of a 50% slurry of protein A agarose (Invitrogen). The clear lysates were incubated with 40 μl of a 50% slurry of protein A beads containing HBc antibody (B0586, Dako) or mock antibody. The immunoprecipitated proteins were separated by 10%, 12%, or 15% SDS-PAGE and immunoblotted using the corresponding antibodies.

### Quantification of secreted HBV DNA copies

To measure secreted HBV DNA, increasing concentrations of Bay41-4109 or SBA_R01 were incubated with HepAD38 cells. The medium was refreshed every 2 d, and HBV DNA in the cell culture medium was quantified at 6 d after treatment. The culture supernatants were treated with DNase I for 1 h at 37°C and incubated at 75°C for 10 min. The supernatants were centrifuged at 10,000 ×g, and the pellets were discarded. The DNA was extracted from the supernatants using a QiAamp DNA mini Qiagen kit (Qiagen) and quantified with a Hepatitis B Viral DNA Quantitative Fluorescence Diagnostic Kit (Santurebiotech, China) according to manufacturer’s instructions. For HBV-transgenic mice, the serum was directly quantified using a Hepatitis B Viral DNA Quantitative Fluorescence Diagnostic Kit (Santurebiotech, China).

### Density gradient analysis

Density gradient analysis was carried out as previously described [17] with slight modification. The cells were lysed in 2 ml of cell lysis buffer (10 mM Tris-HCl pH 8.0, 1 mM EDTA, 0.5% NP40) at room temperature for 10 min. The cell lysates were cleared by centrifugation at 10,000 ×g for 10 min at 4°C. After ultrafiltration of the supernatant with an Amicon centrifugal filter (100-KDa cutoff; Sigma, UFC510008), condensed samples were loaded on a iodixanol density gradient (5%–55%; Sigma, D1556) in cell lysis buffer and then centrifuged at 220,000 ×g (Hitachi CS120FNX, S52ST) for 16 h. Each fraction (0.4 ml/fraction) was collected from the centrifuge tube. For each fraction, 20 μl was subjected to dot blotting to detect HBc protein with HBc antibody (DAKO, B0586) and HBV nucleic acid with a biotin-labeled HBV DNA probe. For quantification of pre-core/pgRNA, RNA from each fraction was extracted with TRIzol reagent (Invitrogen). After the digestion of contaminated DNA with RNase-free DNase I (Thermo, M6101), pre-core/pgRNA was reverse transcripted and quantified with qPCR using paired forward and reverse primers: 5’-GAGTGTGGATTCGCACTCC-3’ and 5’-GAGGCGAGGGAGTTCTTCT-3’. For the negative control, RNA extracted from the 8^th^ fraction of the DMSO-treated cell lysates was directly subjected to qPCR without reverse transcription. The capsid and aberrant polymer-containing fractions were evaluated by transmission electron microscopy (FEI Tecnai G2) after negative staining with uranyl acetate.

### Native agarose gel assay for intracellular capsid or aberrant polymers

The intracellular HBV capsids were analyzed using the previously described procedure [50]. Briefly, the cells or liver were lysed NP-40 buffer (0.1% NP40, 1 mM EDTA, 100 mM NaCl, and 10 mM Tris-HCl; pH 7.6), and core particles were obtained by 7% PEG8000 precipitation. The amount of assembled capsid particles was determined by 1.5% agarose gel electrophoresis. The particles were transferred to a nitrocellulose membrane and detected with polyclonal anti-HBc (Dako, B0586, 1:800 dilution). For Bay41-4109-induced aberrant polymers, the 5^th^ and 8^th^ fractions of density gradient centrifugation assay were subject to 1.5% agarose gel electrophoresis and detected by anti-HBc (Abcam, 8637).

### HBV infection

HBV infection was carried out as previously described[51] with slight modification. Briefly, HepG2 cells expressing human Na^+^-taurocholate cotransporting polypeptide (HepG2-NTCP-2B1 cell line)[51] were pretreated with 2% DMSO for 2 d to arrest cell growth. HepG2-NTCP cells were infected with HBV virions collected from the culture supernatant of HepAD38 cells at a multiplicity of infection (MOI) of 500 viral genome equivalents (VGE) per cell in the presence of 2% DMSO and 4% PEG 8000 followed by treatment with 1 μM Bay41-4109. At 12 h after incubation, the cells were washed with PBS and cultivated in DMEM medium in the presence of 1 μM Bay41-4109. The culture supernatant was collected for the quantification of HBeAg and HBV DNA every other day until day six. The cells were then subjected to immunofluorescence staining with anti-HBc.

### Hirt DNA extraction and cccDNA quantification

HBV cccDNA were extracted from HBV-infected HepG2-NTCP cells by a Hirt DNA extraction procedure. The hirt extrated DNA was digested with plasmid-safe adenosine triphosphate (ATP)-dependent deoxyribonuclease (PSAD) (Epicentre Technologies) to remove rcDNA. PSAD was inactivated by heating at 70°C for 30 min. Subsequently, the level of cccDNA in the Hirt extracted, PSAD-treated samples were quantified by a real time PCR assay with primer sequences GGGGCGCACCTCTCTTTA (forward) and CCACCCAGGTAGCTAGAGTCATTAG (reverse).

### Animal experiments

HBV transgenic mice were selected for age (eight weeks) and sex (male) along with the serum levels of HBV DNA (10^6^–10^7^ IU/ml) and HBeAg (25–50 S/CO) prior to the experiments.

Adenovirus-associated virus 8 (AAV8) was used to achieve the overexpression of human STUB1 in the liver. High-titer AAV8 particles were produced and supplied by Hanbio Biotechnology (Shanghai, China). Viral genomes of AAV8-STUB1 or AAV8-GFP (2 × 10^11^) in 100 μl of PBS were intravenously injected into HBV transgenic mice. At 30 d after injection, the mice were sacrificed for the quantification of serum HBV DNA and HBc expression. Alternatively, at 21 d after injection, the mice were treated with Bay41-4109 or dissolvent control.Mice were randomized into five groups treated with: (i) vehicle and AAV8-GFP (control) (n = 7); (ii) vehicle and AAV8-STUB1 (n = 6); (iii) Bay41-4109 and AAV8-GFP (n = 8); (iv) Bay41-4109 and AAV8-STUB1 (n = 8); and (v) entecavir (n = 6). The mice were administered with AAV8-STUB1 or AAV8-GFP (i.v.) (1×10^11^ virus genome equivalents/mouse). At three weeks after injection, 10 mg/kg Bay41-4109 or vehicle was administrated intraperitoneally twice a day. For group (v), daily oral entecavir (3.2 mg/kg) was applied. Orbital blood samples were taken every 6 d to monitor HBV titer, HBeAg, and HBsAg. Thirty days after the first injection of chemicals, the mice were sacrificed by cervical dislocation. The levels of intracellular HBc and STUB1 were detected by western blot and IHC.

### Detection of serum HBsAg and HBeAg and IHC analysis

HBsAg were detected quantitatively by Architect assay (Abbott Laboratories, Chicago, IL). Liver tissues were dehydrated, embedded in paraffin, and cut into slices with 3-mm thickness for immunohistological analysis. HBcAg and FLAG-tagged STUB1 were stained with polyclonal anti-HBc (Dako, B0586, 1:300 dilution) and anti-FLAG STUB1 (Cell Signaling, 2368, 1:500 dilution), respectively.

HBeAg from cell culture supernatant of HepAD38 cells and HBV-infected HepG2-NTCP cells were measured as the procedure described previously[41]. The monoclonal antibody 16D9, which was used for specificly isolating HBeAg or precore protein, was kindly gifted by Professor Xia Ningshao (Xia Men University). For the HBeAg in HBV transgenic mice were detected quantitatively by Architect assay (Abbott Laboratories, Chicago, IL).

## Supporting information

S1 Fig The knock-down of CUL5, MDM2, PRKN do not restore the HBc protein level in Bay41-4109-treated cells. (A, B) HepAD38 cells were transfected with two different siRNAs targeting CUL5, MDM2, PRKN or mock siRNA. At 2 d after transfection, the cell lysates were detected by western blot using the indicated antibodies. HBc protein levels normalized to actin levels were quantified (A, upper panel). The quantification results of HBc/actin ratio from two independent immunoblots are shown as relative percentages (right panel). The samples of mock treatment were set to 100%. The error bars indicate ±SD. n. s. indicates *p* > 0.05. *p* was calculated by unpaired two-tailed student’s t-test (A, lower panel). The knock-down of E3 ligase were validated by rt-qPCR (B).
(TIF)

S2 Fig The effects of over-expression and siRNA-mediated silencing of STUB1 on the HBc protein level. (A) HepAD38 cells were transfected with two different siRNAs targeting STUB1 or mock siRNA. (B) HepAD38 cells were transfected with pcDNA3.1-STUB1 or control plasmid. (A, B) At 2 d after transfection, the cell lysates were detected by western blot using the indicated antibodies (upper panel). (C) HepAD38 cells were transduced with LV-STUB1 or LV-control. At 36 h after transduction, cells were treated with 50 μg/ml CHX for the indicated time. The proteins were detected by western blot using the indicated antibodies(upper panel). (A, B, C, lower panel) The quantification results of three independent immunoblots are shown as relative percentages (HBc/Actin) with mock transfection/transduction samples set to 100%. The error bars indicate ±SD. Data were analyzed by one-way analysis of variance, followed by Tukey’s comparison test for all groups * indicates p < 0.05. n. s. indicates p > 0.05.
(TIF)

S3 Fig Representative electron micrographs of the indicated fractions from iodixanol gradient centrifugation. Indicated franctions of DMSO- or Bay41-4109-treated samples from the Fig 2 were stained with uranyl acetate and evaluated by transmission electron microscopy.
(TIF)

S4 Fig Particle gel assay of Bay41-4109-induced aberrant polymers. Indicated franctions of DMSO- or Bay41-4109-treated samples from the Fig 2 were subjected to particle gel assay.
(TIF)

S5 Fig Proteasome inhibitors had no effect on HBc protein level in Bay41-4109-treated cells. (A, B) HepAD38 cells were treated with 1 μM Bay41-4109 or DMSO for 24 h, followed by treatment with the proteasome inhibitor lactacystin, lysosome inhibitor BafA1, or lysosome inhibitor Pep/E64 for another 24 h. Cell extracts were then analyzed by western blotting using the indicated antibodies (A). The quantification results of two independent immunoblots are shown as relative percentages (HBc/actin) with the DMSO-treated sample set to 100% (B). The error bars indicate ±SD. n. s. indicates *p* > 0.05, *p* value were calculated by unpaired two-tailed student’s t-test.
(TIF)

S6 Fig Bay41-4109 does not alter the ubiquitylation of HBc. HepAD38 cells were co-transfected with ubiquitin or ubiquitin and STUB1 followed by treatment with Bay41-4109 alone or Bay41-4109 and Baf A1 as indicated. The ubiquitylation of HBc was analyzed by immunoprecipitation followed by SDS-PAGE and western blot.
(TIF)

S7 Fig HSP70 processes Bay41-4109-induced aberrant polymers. (A) HepAD38 cells were transfected with two different siRNAs targeting Hsp70 or mock siRNA. At 2 d after transfection, the cell lysates were detected by western blot using the indicated antibodies. (B) HBc protein levels normalized to actin levels were quantified. The quantification results of HBc/actin ratio from two independent immunoblots are shown as relative percentages. (C) HepAD38 cells were treated with Bay41-4109 or DMSO or Bay41-4109 and MKT077 for 48 h. Cell extracts were then analyzed by western blotting using the indicated antibodies. (D) HBc protein levels normalized to actin levels were quantified. The quantification results of HBc/actin ratio from two independent immunoblots are shown as relative percentages.
(TIF)

S8 Fig STUB1 has no significant influence on the EC50 of SBA_R01 and the lysosome localization of HBc. (A) HepAD38 cells were infected with LV-STUB1 or LV-contr. At 24 h after infection, the cells were treated with the indicated concentration of SBA_R01 for 72 h. Secreted HBV DNA was quantified by qPCR. (B) HepAD38 cells were treated with 1 μM SBA_R01 or DMSO as indicated for 2 d followed by treatment with 0.1 mM BafA1 for 12 h. The cells were immunostained for HBc (green) and LAMP1 (red). Nuclei were stained with Hochest33342. Areas indicated by white boxes are enlarged. Arrows point to typical co-localized sites. Scale bar is 10 μM.
(TIF)

S9 Fig STUB1 does not reduce HBsAg in the presence of Bay41-4109. HepAD38 cells infected with LV-STUB1 or LV-contr. were treated with the indicated concentration of Bay41-4109 for 6 d. Media were refreshed every 2 d. The HBsAg levels in the media were quantified.
(TIF)

S10 Fig Effect of STUB1 overexpression on the viability of LV-STUB1-transduced HepG2-NTCP cells. HepG2-NTCP cells were transduced with LV-STUB1 or LV-control, and treated with Bay41-4109 or DMSO. Cell viability were tested by CCK8 assay.
(TIF)

S11 Fig STUB1 overexpression enhances the Bay41-4109-induced reduction of HBc proteins in HBV-infected HepG2-NTCP cells. (A, B) HepG2-NTCP cells transduced with LV-STUB1 or LV-control were infected with HBV at MOI of 500 genome equivalents in the presence of 2% DMSO. Bay41-4109 (1 μM) or DMSO was added during HBV infection. 6 d after HBV infection, cell extracts were then analyzed by western blotting using indicated antibodies (A). HBc protein levels normalized to actin levels were quantified. The quantification results of HBc/actin ratio from two independent immunoblots are shown as relative percentages (B).
(TIF)

S12 Fig STUB1 knockdown increases the Bay41-4109-induced foci of HBc proteins in HBV-infected HepG2-NTCP cells. (A, B, C) HepG2-NTCP cells transduced with LV-sh_STUB1 or LV-sh_control were infected with HBV at MOI of 500 genome equivalents in the presence of 2% DMSO. Bay41-4109 (1 μM) or DMSO was added during HBV infection. 6 d after HBV infection, cells were immunostained for HBc (green) as indicated. Nuclei were stained with Hochest33342 (A) The fluorescence signal intensity was quantified by ImageJ (B). *p < 0.05, *p* value was calculated by unpaired two-tailed student’s t-test. The knockdown of stub1 were verified by western blot (C).
(TIF)

S13 Fig STUB1 has no significant effect on HBsAg in HBV transgenic mice. HBV transgenic mice were treated as described in Fig 8. The level of HBsAg in serum was quantified every 6 d.
(TIF)
