# Supplementary figures and images for: Bay41-4109-induced aberrant polymers of hepatitis b capsid proteins are removed via STUB1-promoted p62-mediated macroautophagy
Source: PLoS Pathog. 2022 Jan 14;18(1):e1010204. doi: 10.1371/journal.ppat.1010204 (PMC8824320; doi:10.1371/journal.ppat.1010204)

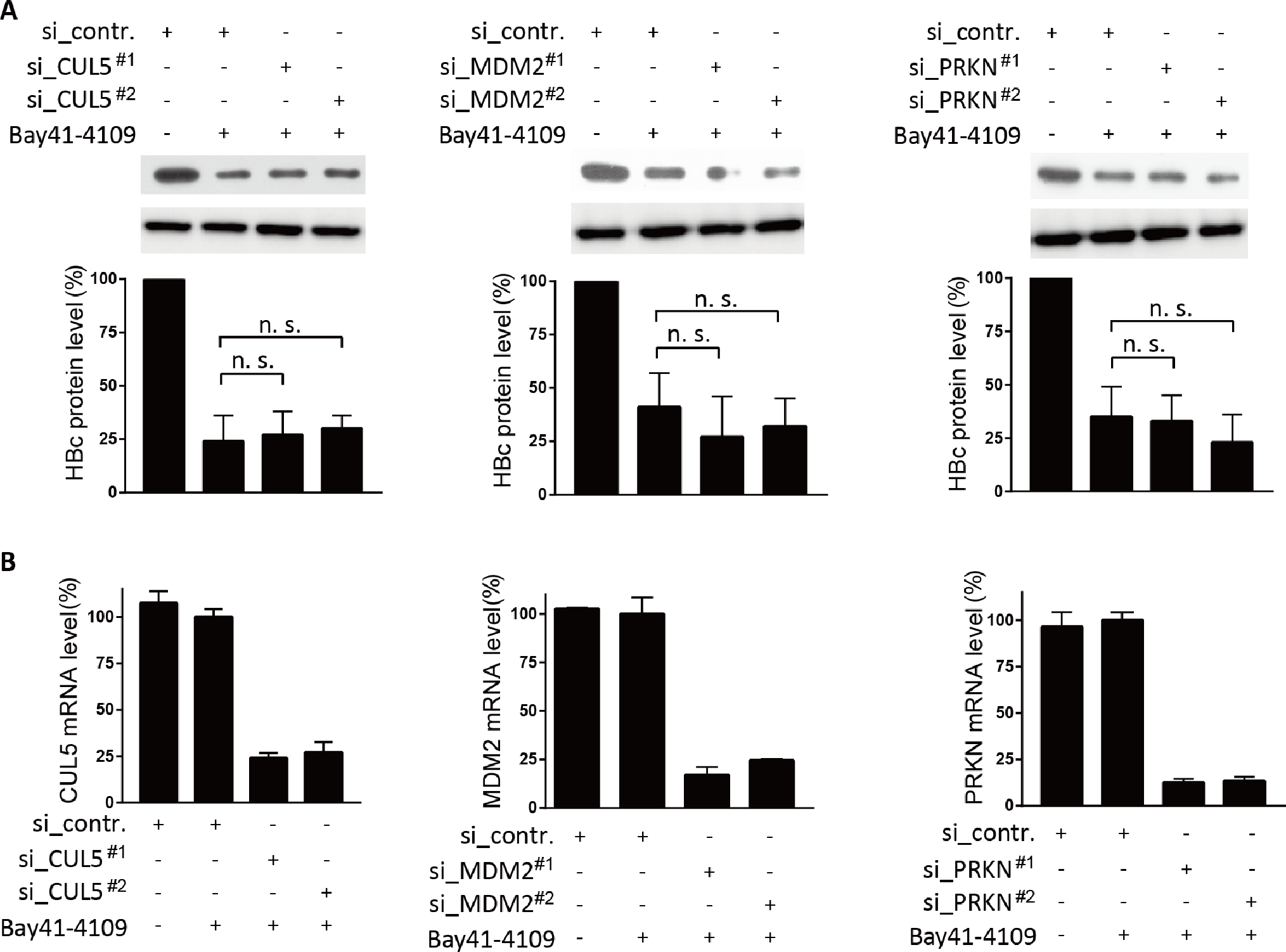

Supplement: S1 Fig — (A, B) HepAD38 cells were transfected with two different siRNAs targeting CUL5, MDM2, PRKN or mock siRNA. At 2 d after transfection, the cell lysates were detected by western blot using the indicated antibodies. HBc protein levels normalized to actin levels were quantified (A, upper panel). The quantification results of HBc/actin ratio from two independent immunoblots are shown as relative percentages (right panel). The samples of mock treatment were set to 100%. The error bars indicate ±SD. n. s. indicates p > 0.05. p was calculated by unpaired two-tailed student’s t-test (A, lower panel). The knock-down of E3 ligase were validated by rt-qPCR (B). (TIF) [file ppat.1010204.s001.tif]

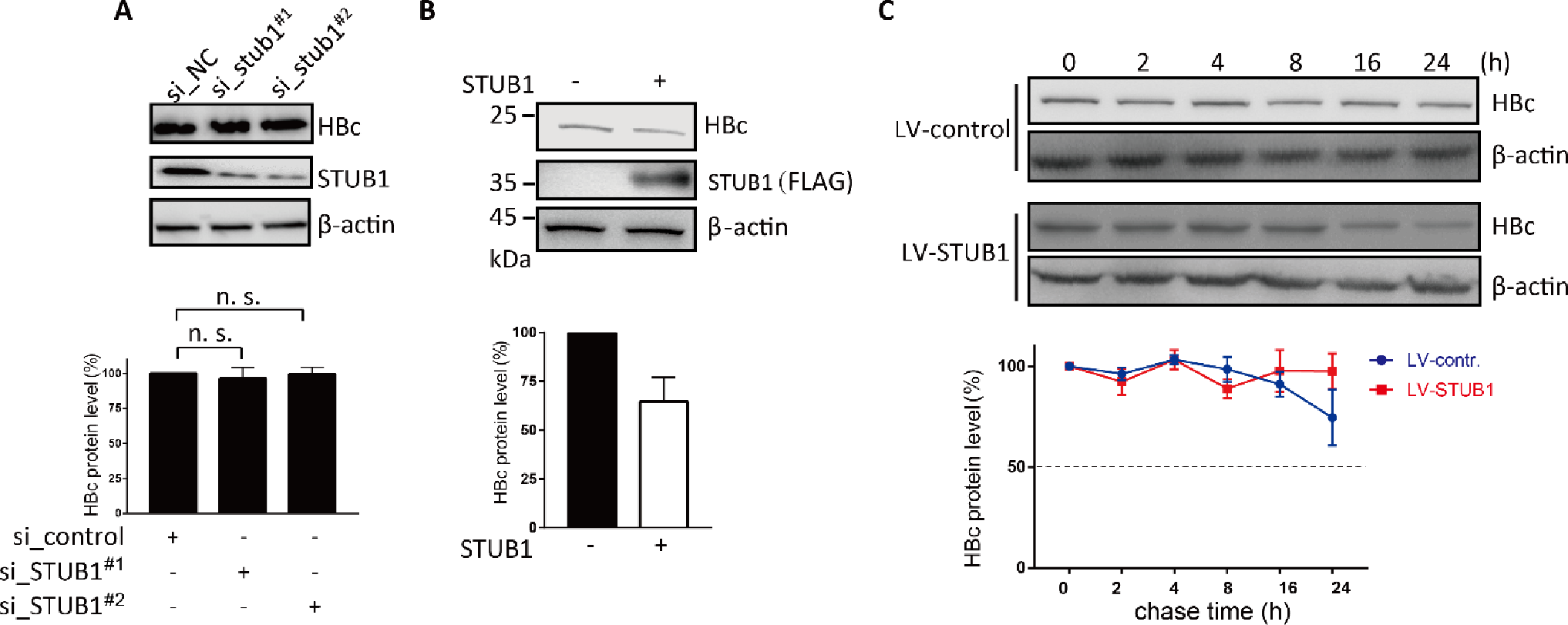

Supplement: S2 Fig — (A) HepAD38 cells were transfected with two different siRNAs targeting STUB1 or mock siRNA. (B) HepAD38 cells were transfected with pcDNA3.1-STUB1 or control plasmid. (A, B) At 2 d after transfection, the cell lysates were detected by western blot using the indicated antibodies (upper panel). (C) HepAD38 cells were transduced with LV-STUB1 or LV-control. At 36 h after transduction, cells were treated with 50 μg/ml CHX for the indicated time. The proteins were detected by western blot using the indicated antibodies(upper panel). (A, B, C, lower panel) The quantification results of three independent immunoblots are shown as relative percentages (HBc/Actin) with mock transfection/transduction samples set to 100%. The error bars indicate ±SD. Data were analyzed by one-way analysis of variance, followed by Tukey’s comparison test for all groups * indicates p < 0.05. n. s. indicates p > 0.05. (TIF) [file ppat.1010204.s002.tif]

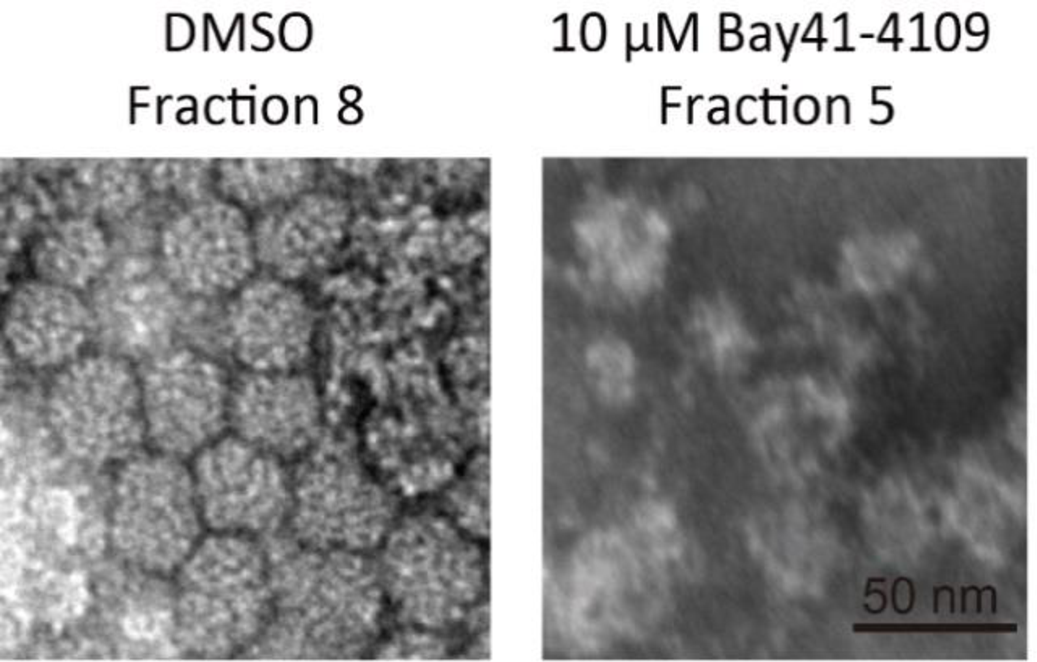

Supplement: S3 Fig — Indicated franctions of DMSO- or Bay41-4109-treated samples from the Fig 2 were stained with uranyl acetate and evaluated by transmission electron microscopy. (TIF) [file ppat.1010204.s003.tif]

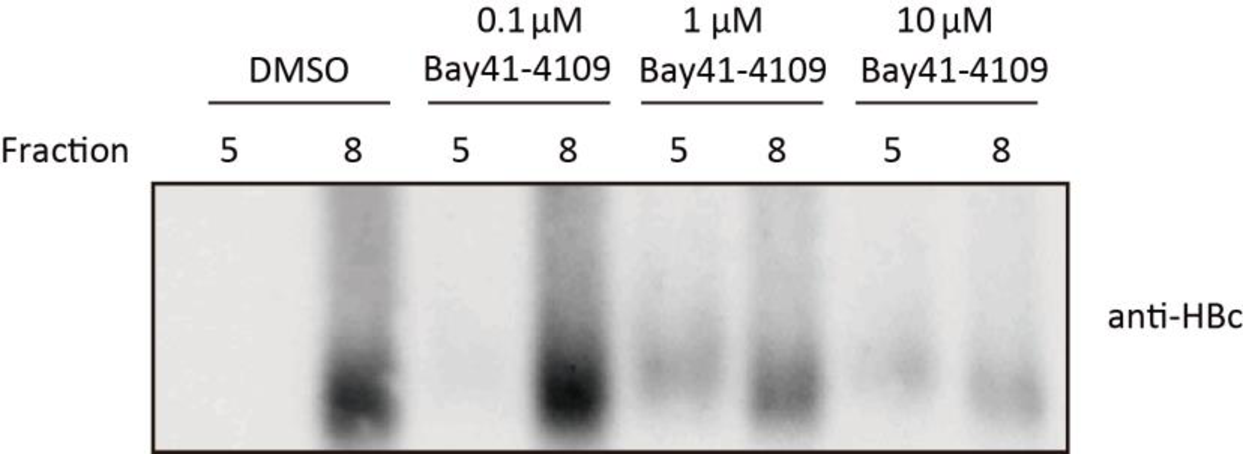

Supplement: S4 Fig — Indicated franctions of DMSO- or Bay41-4109-treated samples from the Fig 2 were subjected to particle gel assay. (TIF) [file ppat.1010204.s004.tif]

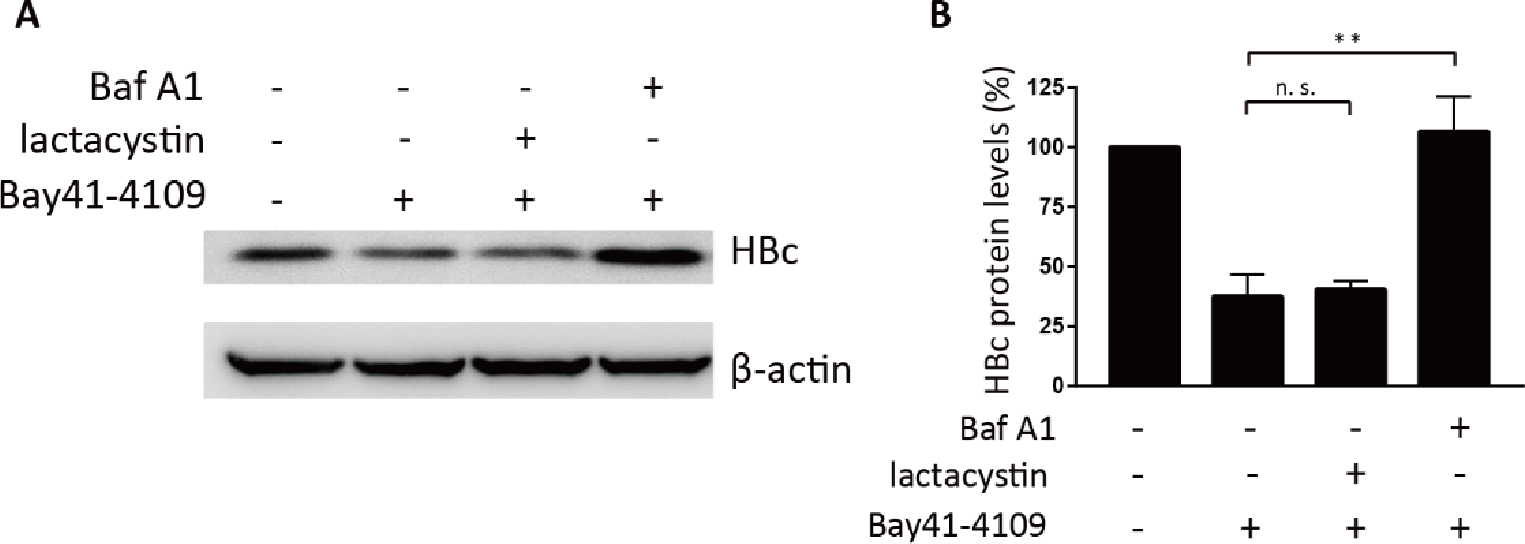

Supplement: S5 Fig — (A, B) HepAD38 cells were treated with 1 μM Bay41-4109 or DMSO for 24 h, followed by treatment with the proteasome inhibitor lactacystin, lysosome inhibitor BafA1, or lysosome inhibitor Pep/E64 for another 24 h. Cell extracts were then analyzed by western blotting using the indicated antibodies (A). The quantification results of two independent immunoblots are shown as relative percentages (HBc/actin) with the DMSO-treated sample set to 100% (B). The error bars indicate ±SD. n. s. indicates p > 0.05, p value were calculated by unpaired two-tailed student’s t-test. (TIF) [file ppat.1010204.s005.tif]

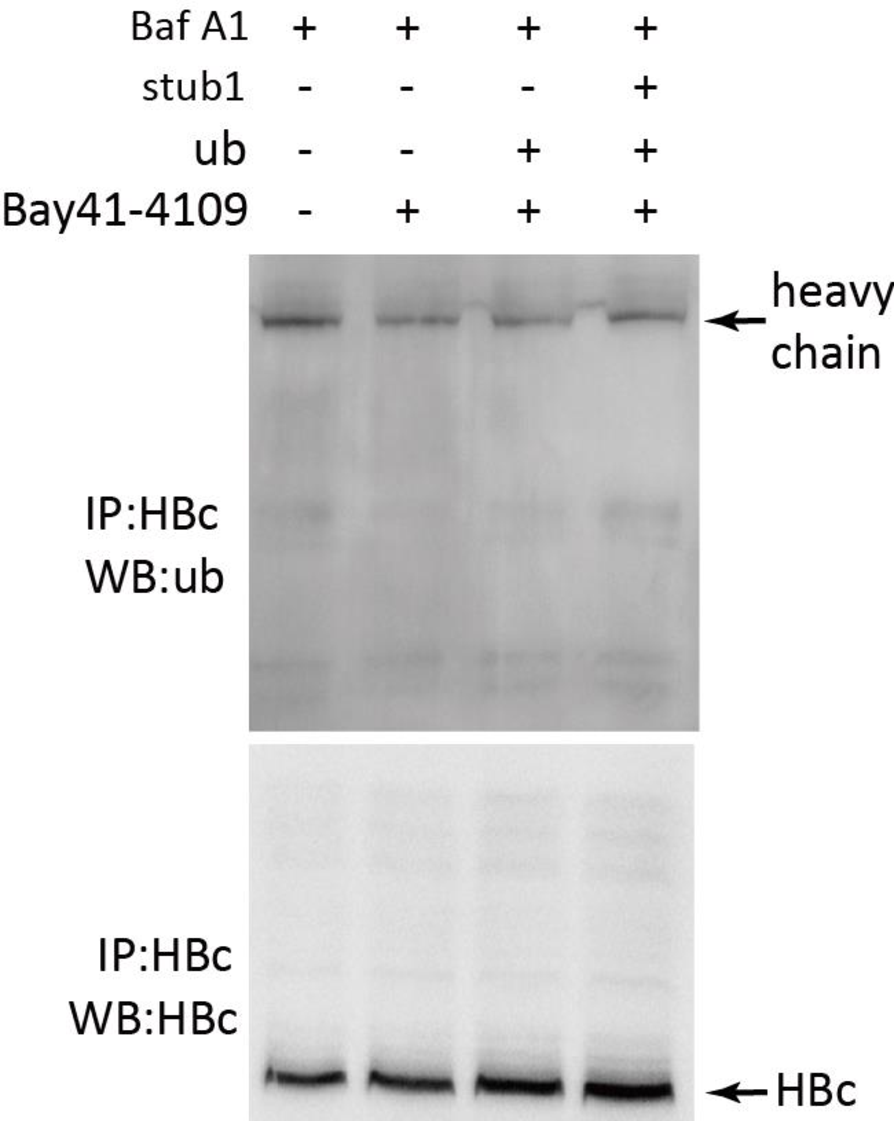

Supplement: S6 Fig — HepAD38 cells were co-transfected with ubiquitin or ubiquitin and STUB1 followed by treatment with Bay41-4109 alone or Bay41-4109 and Baf A1 as indicated. The ubiquitylation of HBc was analyzed by immunoprecipitation followed by SDS-PAGE and western blot. (TIF) [file ppat.1010204.s006.tif]

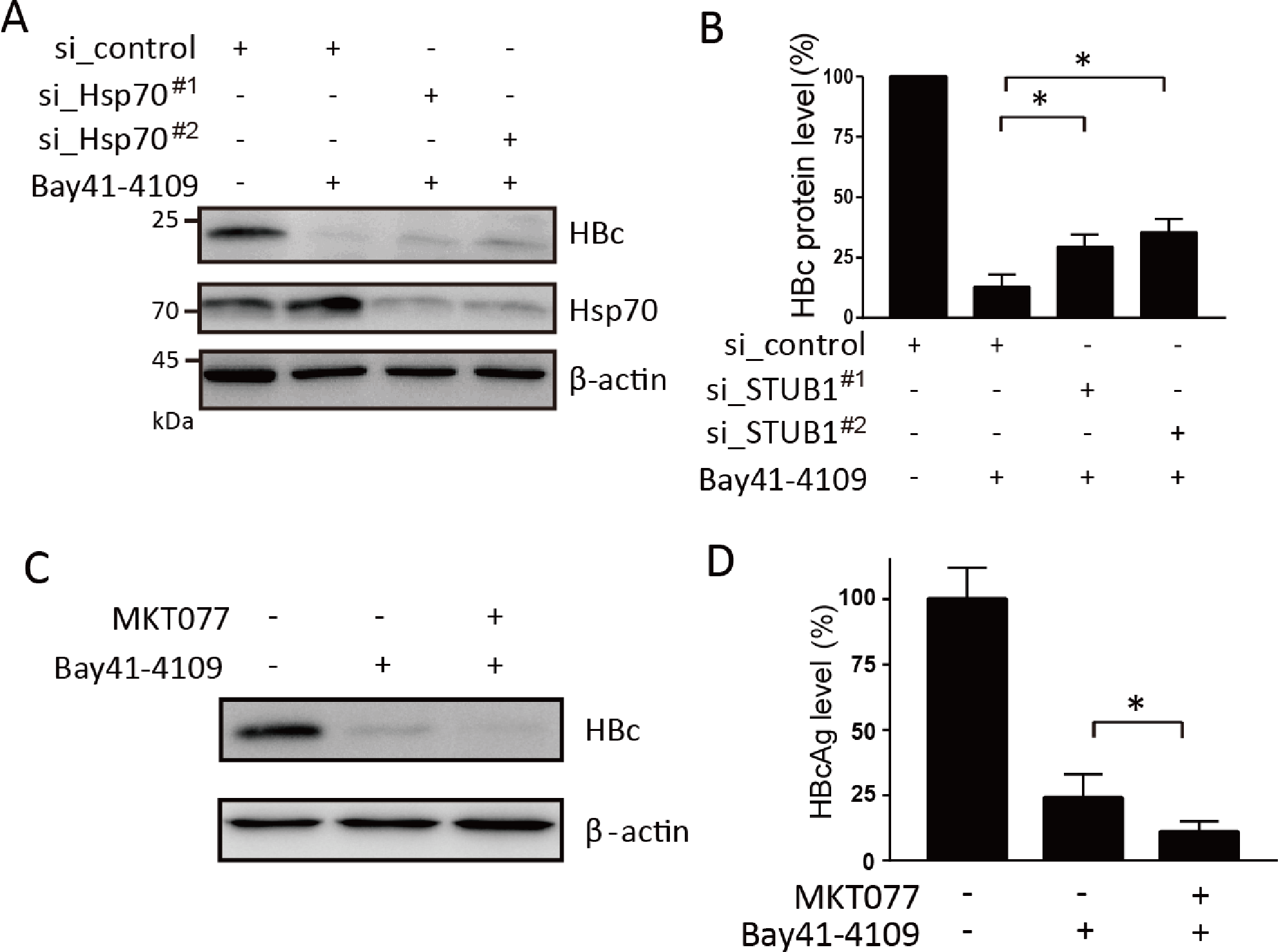

Supplement: S7 Fig — (A) HepAD38 cells were transfected with two different siRNAs targeting Hsp70 or mock siRNA. At 2 d after transfection, the cell lysates were detected by western blot using the indicated antibodies. (B) HBc protein levels normalized to actin levels were quantified. The quantification results of HBc/actin ratio from two independent immunoblots are shown as relative percentages. (C) HepAD38 cells were treated with Bay41-4109 or DMSO or Bay41-4109 and MKT077 for 48 h. Cell extracts were then analyzed by western blotting using the indicated antibodies. (D) HBc protein levels normalized to actin levels were quantified. The quantification results of HBc/actin ratio from two independent immunoblots are shown as relative percentages. (TIF) [file ppat.1010204.s007.tif]

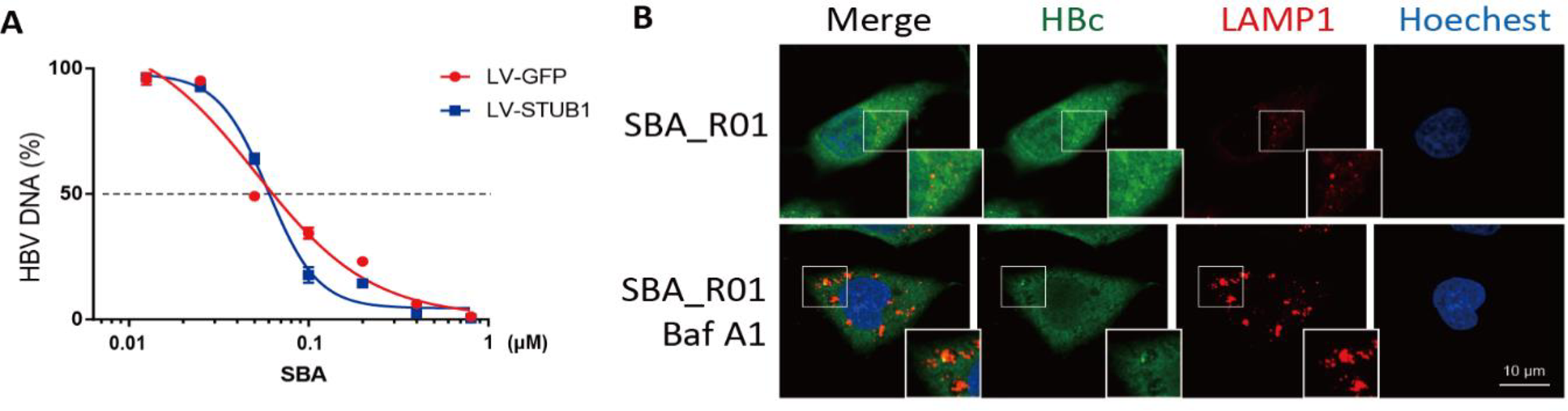

Supplement: S8 Fig — (A) HepAD38 cells were infected with LV-STUB1 or LV-contr. At 24 h after infection, the cells were treated with the indicated concentration of SBA_R01 for 72 h. Secreted HBV DNA was quantified by qPCR. (B) HepAD38 cells were treated with 1 μM SBA_R01 or DMSO as indicated for 2 d followed by treatment with 0.1 mM BafA1 for 12 h. The cells were immunostained for HBc (green) and LAMP1 (red). Nuclei were stained with Hochest33342. Areas indicated by white boxes are enlarged. Arrows point to typical co-localized sites. Scale bar is 10 μM. (TIF) [file ppat.1010204.s008.tif]

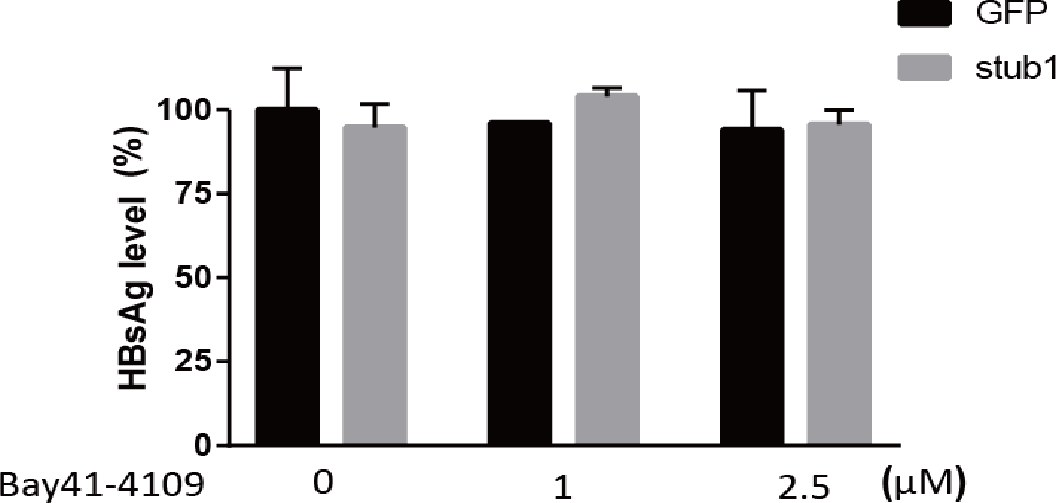

Supplement: S9 Fig — HepAD38 cells infected with LV-STUB1 or LV-contr. were treated with the indicated concentration of Bay41-4109 for 6 d. Media were refreshed every 2 d. The HBsAg levels in the media were quantified. (TIF) [file ppat.1010204.s009.tif]

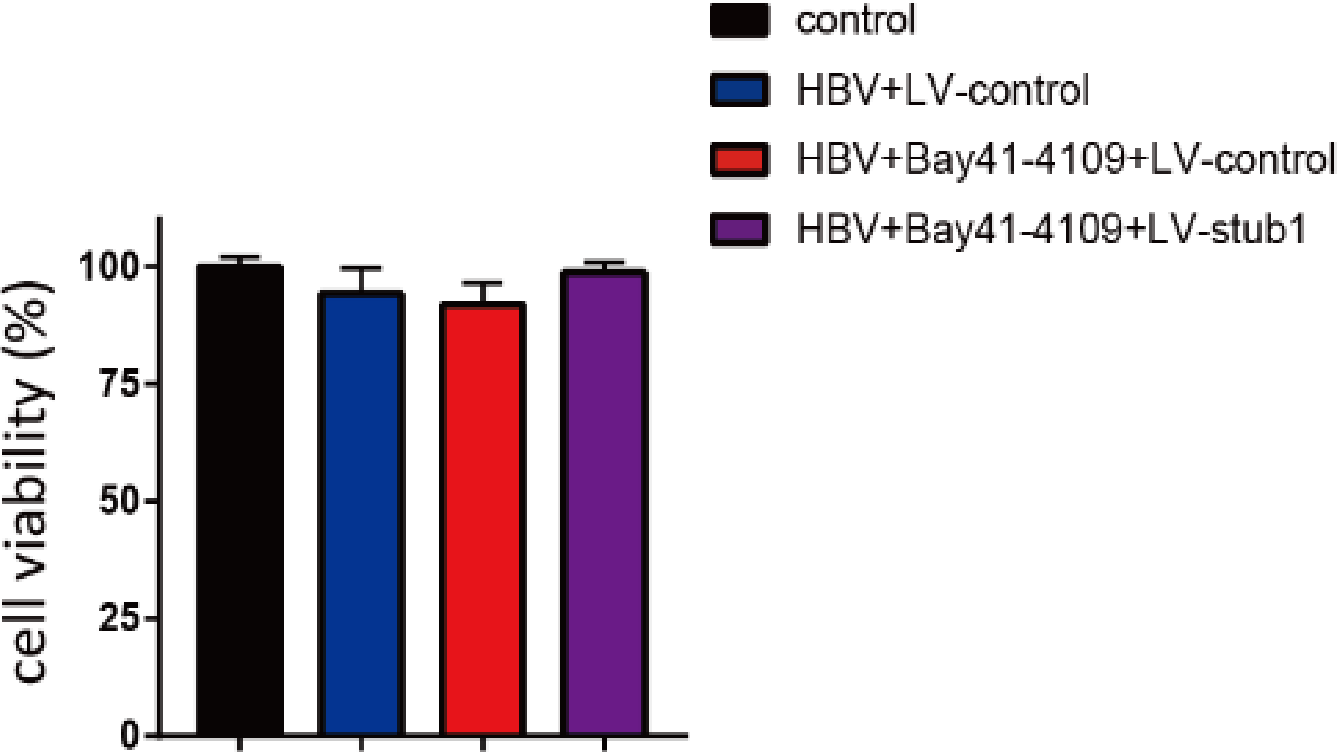

Supplement: S10 Fig — HepG2-NTCP cells were transduced with LV-STUB1 or LV-control, and treated with Bay41-4109 or DMSO. Cell viability were tested by CCK8 assay. (TIF) [file ppat.1010204.s010.tif]

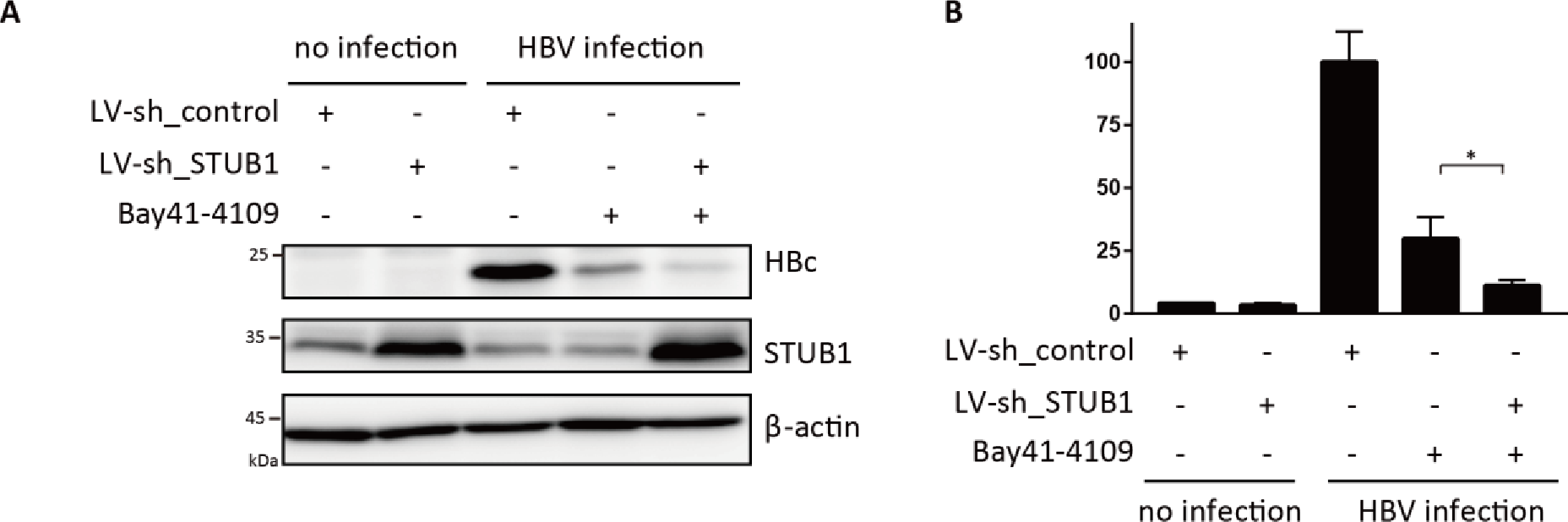

Supplement: S11 Fig — (A, B) HepG2-NTCP cells transduced with LV-STUB1 or LV-control were infected with HBV at MOI of 500 genome equivalents in the presence of 2% DMSO. Bay41-4109 (1 μM) or DMSO was added during HBV infection. 6 d after HBV infection, cell extracts were then analyzed by western blotting using indicated antibodies (A). HBc protein levels normalized to actin levels were quantified. The quantification results of HBc/actin ratio from two independent immunoblots are shown as relative percentages (B). (TIF) [file ppat.1010204.s011.tif]

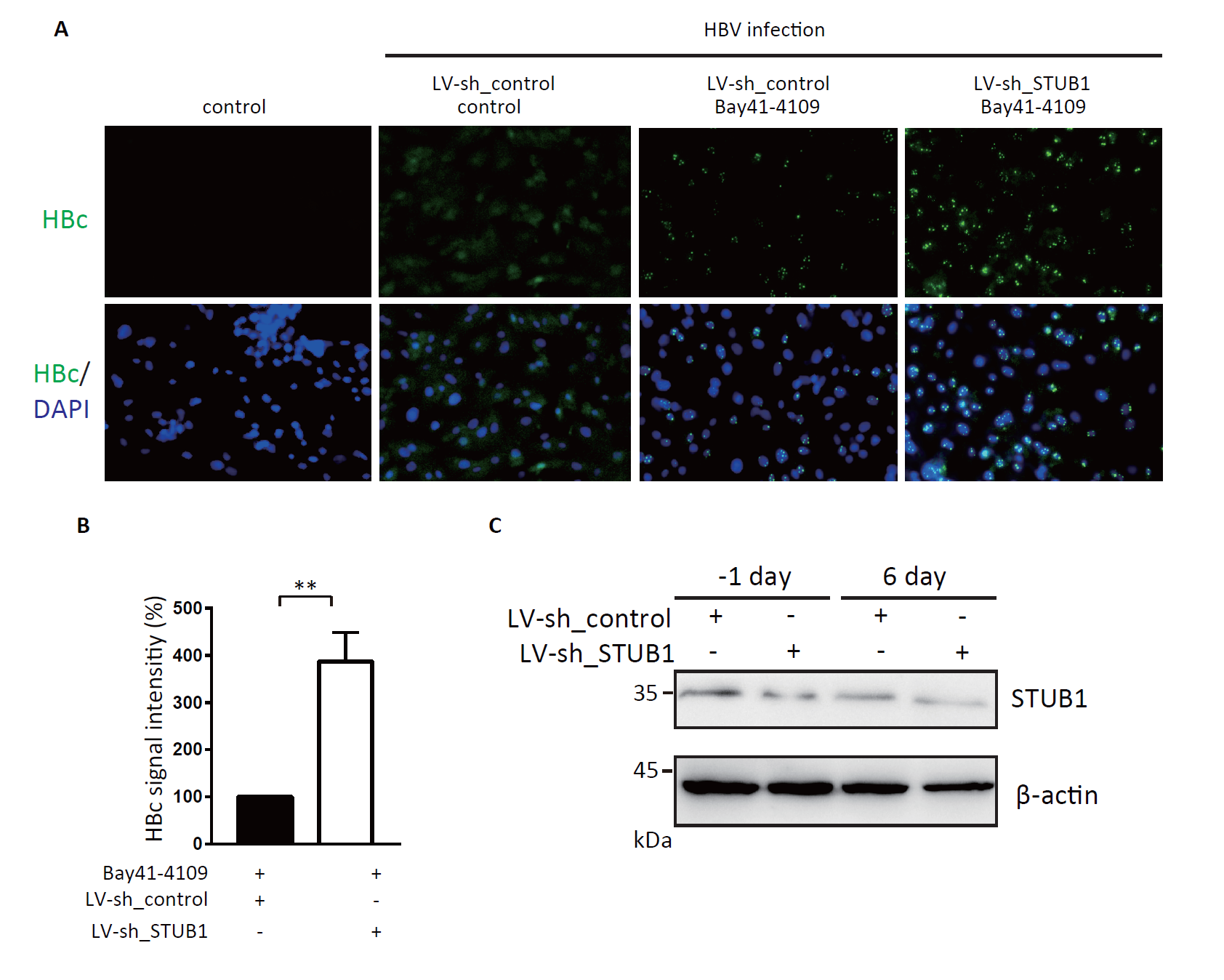

Supplement: S12 Fig — (A, B, C) HepG2-NTCP cells transduced with LV-sh_STUB1 or LV-sh_control were infected with HBV at MOI of 500 genome equivalents in the presence of 2% DMSO. Bay41-4109 (1 μM) or DMSO was added during HBV infection. 6 d after HBV infection, cells were immunostained for HBc (green) as indicated. Nuclei were stained with Hochest33342 (A) The fluorescence signal intensity was quantified by ImageJ (B). *p < 0.05, p value was calculated by unpaired two-tailed student’s t-test. The knockdown of stub1 were verified by western blot (C). (TIF) [file ppat.1010204.s012.tif]

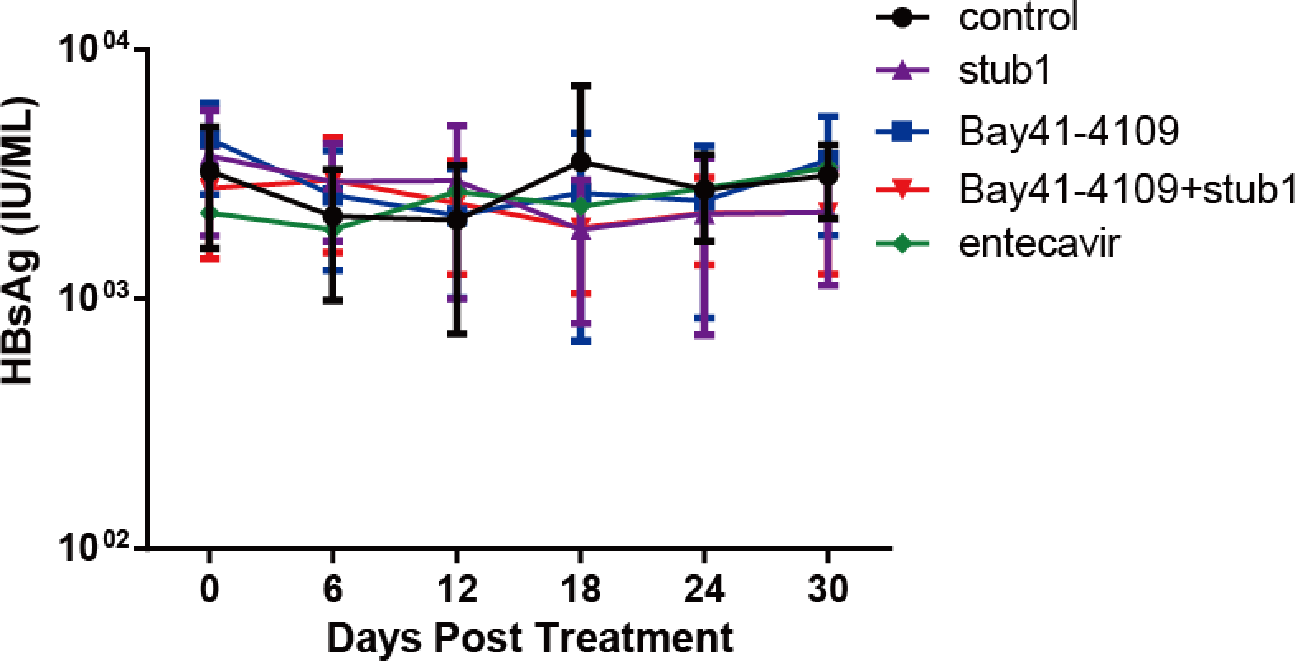

Supplement: S13 Fig — HBV transgenic mice were treated as described in Fig 8. The level of HBsAg in serum was quantified every 6 d. (TIF) [file ppat.1010204.s013.tif]
